# The interplay of gut microbiota between donors and recipients determines the efficacy of fecal microbiota transplantation

**DOI:** 10.1080/19490976.2022.2100197

**Published:** 2022-07-19

**Authors:** Ruiqiao He, Pan Li, Jinfeng Wang, Bota Cui, Faming Zhang, Fangqing Zhao

**Affiliations:** a Beijing Institutes of Life Science, Chinese Academy of Sciences, Beijing, China; b University of Chinese Academy of Sciences, Beijing, China; c Medical Center for Digestive Diseases, the Second Affiliated Hospital of Nanjing Medical University, Nanjing, Jiangsu, China; d Key Laboratory of Holistic Integrative Enterology, Nanjing Medical University, Nanjing, Jiangsu, China; e Center for Excellence in Animal Evolution and Genetics, Chinese Academy of Sciences, Kunming, China; f Key Laboratory of Systems Biology, Hangzhou Institute for Advanced Study, University of Chinese Academy of Sciences, Hangzhou, Zhejiang, China

**Keywords:** Enterotype, fecal microbiota transplantation, gut microbiota, washed microbiota transplantation, dysbiosis, ulcerative colitis, Crohn‘s disease

## Abstract

Fecal microbiota transplantation (FMT) is a promising treatment for microbiota dysbiosis associated diseases, such as *Clostridioides difficile* infection (CDI) and inflammatory bowel disease (IBD). The engraftment of donor bacteria is essential for the effectiveness of FMT, which to some extent depends on the matching of donors and recipients. However, how different types of donor-derived bacteria affect FMT efficacy has not been fully dissected. We recruited two longitudinal IBD cohorts of 103 FMT recipients and further analyzed 1,280 microbiota datasets from 14 public CDI and IBD studies to uncover the effect of donor-derived microbiota in recipients. We found that two enterotypes, RCPT/E and RCPT/B (dominated by Enterobacteriaceae and *Bacteroides*, respectively), consistently exist in both CDI and IBD patients. Based on a time-course-based multi-cohort analysis of FMT fecal samples, we observed the interplay between recipient and donor-derived microbiota during FMT, in which the FMT outcome was significantly associated with the enterotype and microbiota distance between donor and recipient after FMT. We proposed a new measurement, the ratio of colonizers to residents after FMT (C2R), to quantify the engraftment of donor-derived bacteria in the recipients, and then constructed an enterotype-based statistical model for donor-recipient matching, which was validated by both cross-validation and an additional IBD FMT cohort (n = 42). We believe that with the accumulation of FMT multi-omics datasets, machine learning-based methods will be helpful for rational donor selection for improving efficacy and precision FMT practices.

## Introduction

Gastrointestinal dysbiosis is closely related to a variety of health problems in humans, of which *Clostridioides difficile* infection (CDI) and inflammatory bowel disease (IBD) are the two most notorious. CDI, caused by the antibiotic resistant gram-positive pathogen *C. difficile*, has a high incidence of ~20% recurrence and results in 30,000 deaths in the US per year.^1^ Meanwhile, IBD affects over 7 million people globally, with symptoms of persistent diarrhea, abdominal pain, and cramping in the gut.^2^ Although CDI and IBD are distinct entities, they are still highly similar in many ways.^3–5^ Recent studies found that both diseases frequently undergo disruption of the gut microbiota.^6,7^ In view of the harmfulness of the two diseases and the defects of conventional therapeutic methods, there is an emerging need for microbial therapy targeting their disordered microbiota.

Fecal microbiota transplantation (FMT) is one of the alternative therapies used enthusiastically in clinical practice in recent years.^8,9^ In FMT, the gut microbiota is transplanted from healthy donors to patients to treat diseases that do not respond to traditional medicine. This technique has proven effective in many microbiota-related metabolic,^10^ infectious,^11^ and inflammatory diseases, including recurrent CDI and IBD. For recurrent CDI, FMT breaks the recurrence cycle and effectively cures up to 85% of patients.^11^ Similarly, the transmitted donor microbes are beneficial for relieving inflammation in IBD patients. In a meta-analysis of 122 patients, the remission rate of IBD after FMT was 45%, which was significantly higher than that in the placebo group (20%).^12^ Unfortunately, although FMT is a feasible solution for treating the two diseases, it is ineffective or associated with relapse in a certain proportion of patients. To improve the efficacy of FMT in precision medicine, understanding the mechanism underlying its effectiveness against the two diseases and the reasons for FMT failure in some cases is necessary.

Screening an effective donor and matching them to a suitable recipient are considered crucial for improving the success rate of FMT. High microbial richness in the donor’s microbiota was found to be associated with the response of IBD patients,^13^ which may be because more microbial species would have more opportunities for engraftment. This donor-dependent effect on FMT efficacy was also reported in many other clinical trials of gastrointestinal disorders, and such donors were recognized as “super-donors”.^14^ Even though super-donors may be a solution for improving the efficacy of FMT, such donors are not easy to identify, and may not fit all.^15^ Therefore, leveraging donor-recipient matching to improve the engraftment of the donor microbiota in the recipient has been taken into consideration and is thought to be essential for FMT success.^16^ Recently, many studies reported that the donor’s microbes were more easily engrafted when certain species were already present in the recipient.^17,18^ However, how donor-recipient matching and engrafted donor microbes contribute to FMT success has not been well elucidated.^19^ Considering that different types of microbial disorders may take place in different diseases, more studies on the principles of effective donor selection and the patterns of donor-recipient matching are still needed.

Here, we firstly recruited a cohort of 61 patients with mild to severe IBD symptoms who completed a full FMT trial to investigate the relationships between their gut microbiota signatures and the FMT response rate. We performed a comprehensive analysis of 1,440 longitudinal microbiota samples from our study and public cohorts of CDI and IBD patients treated with FMT. Next, we explored the gut microbiota signatures through enterotype clustering across the two diseases, quantified donor-derived bacteria that colonized the recipients and tested the contributions of these bacteria to the response rate of FMT. Enterotype matching between recipients and donors that affected the response rate was identified, applied in a clinically accessible machine learning model with rigorous cross-validation, and finally validated in an independent cohort of IBD patients (n = 42) who were recruited in this study and treated with FMT.

## Results

### FMT study design and population characteristics

**Figure 1.**
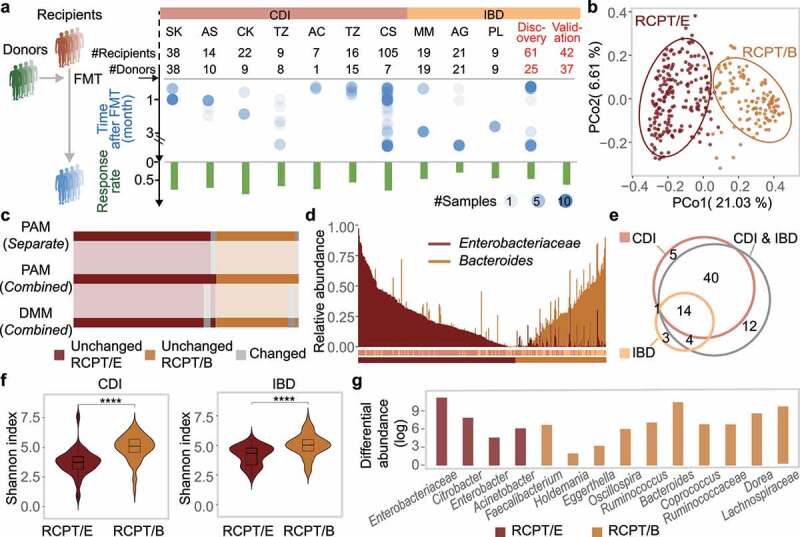
Enterotype analysis in CDI and IBD patients reveals consistency between the two diseases. (a) Outline of cohort collection for FMT to treat CDI and IBD. Only studies that provide detailed metadata about FMT outcomes are depicted. Each column represents one analyzed cohort, denoted by the study’s first author’s abbreviated name. “Discov.” and “Val.” indicate our recruited cohorts for discovery and validation, respectively. “#” and dot size represent the number of sample size in each cohort, blue dots represent samples from recipients after FMT. (b) Bray-Curtis beta-diversity ordination of samples from CDI and IBD patients (n = 322 stool samples). RCPT/E (dark red) and RCPT/B (Orange) are the two enterotypes clustered by enterotype tutorial. (c) Distribution of unchanged and variable individuals among three approaches of enterotype clustering (partitioning around medoids (PAM) separate, PAM combined and Dirichlet multinomial mixtures (DMM) combined). “Unchanged” means that the individual gets the same enterotype with two approaches, and “variable” vice versa. (d) Relative abundance distribution of two enterotype dominant bacteria (RCPT/E and RCPT/B). *Enterobacteriaceae* represents the dominant genus in RCPT/E. (e) The number of enterotype-associated genera in different approaches (Wilcoxon test, q < 0.0001). “CDI” means including only individuals with CDI to obtain enterotype-associated genera, “IBD” means including only IBD, and “CDI & IBD” means both diseases. (f) Alpha diversity between two enterotypes in CDI and IBD. The boxplot center represents median, and the box shows the interquartile range (IQR). Whiskers extend to the most extreme data point < 1.5 x IQR. Asterisks indicate significance (****p < .0001, ***p < .001). (g) Differential abundance of 14 enterotype-characteristic taxa in two enterotypes (Wilcoxon test, q < 0.0001). Differential abundance was calculated by subtracting the mean relative abundance between two enterotypes for each enterotype-characteristic taxon. *Family name* represents the genus for f__Family name; g__. The enterotype-characteristic taxa are sorted according q value. All q value represents the p value adjusted by the Benjamini-Hochberg false discovery rate.

We enrolled 102 IBD patients with mild to severe symptoms with at least a 3-month follow-up evaluation for treatment effectiveness (see Methods and Figure 1a). Sixty-one patients treated by FMT who completed the full trial and had complete fecal samples available were included in the following analyses, with 54.10% and 34.43% of them having moderate and severe IBD symptoms, respectively (Supplementary Table S1). Disease severity has been assessed by clinical activity indices (Harvey–Bradshaw index score or Mayo score) due to clinical practice. Among them, 28 recipients (45.9%) showed symptom relief at the level of disease severity at 3 months, which was designated as a response (see Methods). Mild and moderate adverse events occurred in 31.15% of the recipients, most of whom recovered on their own without medication use in short term, and no serious adverse events occurred during the therapy.

Fecal samples were collected and sequenced from all 61 IBD recipients before and after FMT treatment as well as from their corresponding donors. We further retrieved 16S rRNA amplicon sequencing datasets of longitudinal fecal samples of FMT-treated IBD or CDI patients together with their donors from 14 public cohorts (Supplementary Figure S1a), as CDI is also frequently and effectively treated by FMT.^20–35^ In total, 1,440 amplicon sequencing datasets of IBD and CDI patients were used to explore robust signatures and construct a donor selection model based on donor-recipient matching, and their corresponding characteristics are shown in Supplementary Table S2.

To validate the donor selection model, we further recruited a validation cohort of 42 IBD patients by applying different donor-recipient matching strategies. The disease severity of these patients showed no significant difference from that previously measured (chi-square test, p > .20). Longitudinal fecal samples of these patients before and after FMT as well as from their corresponding donors were collected and sequenced using the same experimental procedure.

### Enterotype analysis in CDI and IBD patients reveals consistency between the two diseases

To make the characteristics of gut microbiota dysbiosis in the 15 IBD and CDI cohorts comparable, we first unified the processing and analysis methods for all microbiome datasets (see Methods and Supplementary Figure S1b-d). After strict quality control, we retained the gut microbial profiles of 322 patients who received FMT (IBD, n = 103; CDI, n = 219), and for each disease, we clustered the patients into different enterotypes. Patients were divided into two enterotypes regardless of disease category (Adonis test, p = .001 for both CDI and IBD) (Supplementary Figure S2). The two enterotypes in different diseases were consistently dominated by Enterobacteriaceae and *Bacteroides* (Wilcoxon test, q < 0.0001), and hereafter, they are denoted as RCPT/E and RCPT/B, respectively (Supplementary Figure S2). In addition, the two enterotypes were pervasive in both CDI and IBD patients with a separate preference (Supplementary Figure S3), implying enterotype is a distinct and reliable typing method for microbiota-related diseases.

To validate the consistency of these two enterotypes in CDI and IBD, we combined all the datasets of the two diseases and performed enterotype clustering using multiple approaches (partitioning around medoids (PAM) and Dirichlet multinomial mixtures (DMM))^36^ As shown in Figure 1b and Supplementary Figure S2, all individuals were still divided into two distinct clusters when using different approaches. More than 90% of the samples were found to be unchanged and to remain in the same cluster across the three clustering approaches, and the clustering results of the combined datasets obtained with PAM were the most stable (Figure 1c). Similarly, the clustering result was also not significantly correlated with the confounding factors, such as “study” or “sequencing methods” (chi-square test, p > .20) (Supplementary Figures S3 and S4), indicating the robustness of enterotype classification for these two diseases.

In addition to measuring the patient composition of the two enterotypes, we further compared the dominant taxa of the two enterotypes using different approaches. As expected, the abundance and prevalence of the two dominant taxa (Enterobacteriaceae and *Bacteroides*) were markedly differentiated across enterotypes (Figure 1d). We further focused on the bacteria that were significantly associated with the enterotype (Wilcoxon test, q < 0.0001) when using different clustering approaches (Figure 1e) and found that 58 out of 67 enterotype-associated bacteria remained significant (Wilcoxon test, q < 0.0001) when merging all samples of CDI and IBD, regardless of the clustering approach (Supplementary Figure S3). In addition, we identified 14 taxa that were shared when analyzing different diseases, which were defined as enterotype-characteristic bacteria (RCPT/E, n = 4; RCPT/B, n = 10). The consistency of enterotype-characteristic bacteria across multiple clustering approaches further demonstrates the stability and reliability of enterotype clustering for these two diseases.

To elucidate why patients suffering from different diseases exhibited similar enterotype clustering, we first measured the associations between enterotype and different clinical factors in patients, and found no significant difference in IBD patients (chi-square test, p > .2, Supplementary Figure S3), except for the factor “corticosteroids history” (chi-square test, p = .02, Supplementary Figure S3h). It indicates that some medications, like corticosteroids, may contribute to shaping patients’ enterotype as RCPT/B. We next explored the microbiota diversity between the two enterotypes, and found that alpha diversity was significantly lower in RCPT/E than in RCPT/B and healthy donors regardless of disease category (Wilcoxon test, p < .005) (Figure 1f), indicating a much more disturbed microbiota in RCPT/E patients. By exploring the differential abundances of the 14 enterotype-characteristic bacteria conserved across diseases, we found that certain bacteria (e.g. *Enterobacter* and *Citrobacter*) tended to be present in the upper gastrointestinal tract, featuring lower pH and higher oxygen levels,^37^ and they were enriched in RCPT/E instead of RCPT/B (Figure 1g), which have been reported to be associated with diarrheal symptoms.^38,39^ Taken together, these results illustrate that enterotype-based analyses can be used to characterize and differentiate the gut microbiota of different IBD and CDI patients.

### FMT outcome was significantly associated with the microbiota distance between recipient and donor

**Figure 2.**
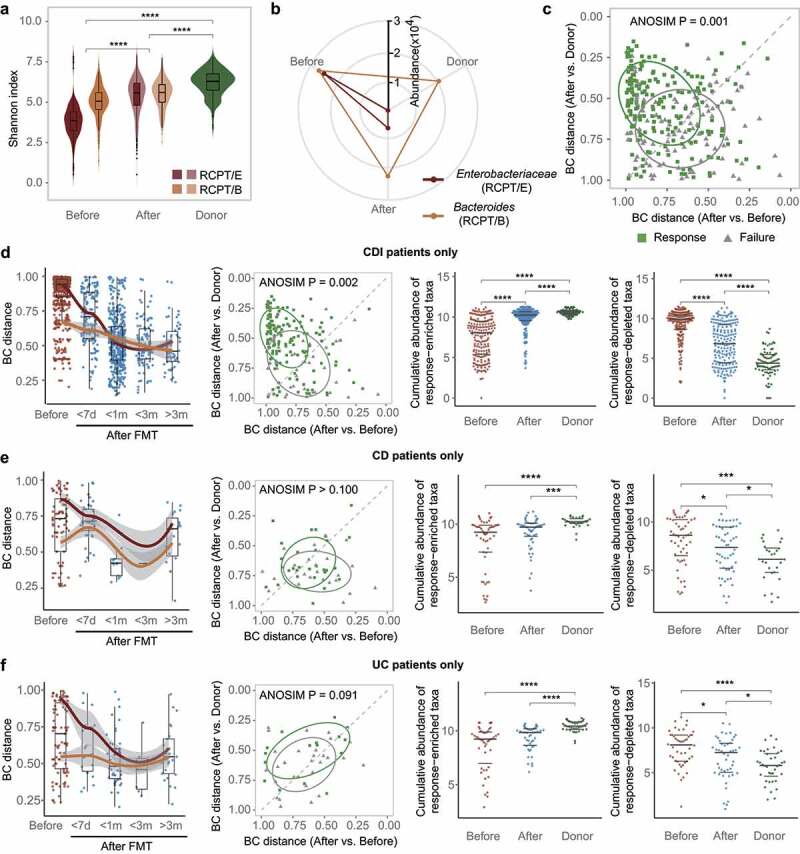
FMT outcome was significantly associated with the microbiota distance between recipient and donor. (a) Comparisons of alpha diversity in individuals with two enterotypes and donors across time in FMT (n = 322). (b) Relative abundance variation of the dominant genus during FMT. *Enterobacteriaceae* represents the dominant genus in RCPT/E. (c) Distribution of recipients in different FMT outcome groups (response and failure) (n = 286). The two coordinates represent the BC distance between the recipient after FMT and the same recipient before FMT or their donor before FMT, respectively. The green and gray points represent the response and failure of FMT, respectively. ANOSIM was performed for the two groups (response and failure) (ANOSIM test, statistic = 0.11, p = .001). (d-f) Analyses were reproduced in CDI recipients (d), IBD subtype CD recipients (e) and IBD subtype UC recipients (f). Left: Community variability was determined by the Bray-Curtis (BC) distance over time during FMT. The red and blue dots represent the BC distance between the recipient and its donor before and after FMT. The two lines fit the trends of RCPT/E and RCPT/B, respectively. Middle: Distribution of recipients in different FMT outcome groups. Right: the cumulative abundance of significantly response-enriched or response-depleted taxa in donors and patients before FMT (Wilcoxon test, q < 0.05). The q value represents the p value adjusted by the Benjamini-Hochberg false discovery rate. Cumulative abundance was calculated by summing all genera that were significantly enriched or depleted in the response group. Asterisks indicate significance (****p < .0001, ***p < .001, **p < .01, *p < .05).

Considering the two types of gut microbiota dysbiosis among the patients suffering from CDI and IBD, we investigated the improvement of the recipient’s gut microbiota after FMT from the perspective of enterotype-based analysis. After FMT, the alpha diversity of the recipient’s gut microbiota increased significantly over time (Wilcoxon test, p < .05), where both enterotypes exhibited a significant increase (Wilcoxon test, p < .005) and approximated the level of healthy donors (Figure 2a and Supplementary Figure S5). Notably, for RCPT/E marked with a dramatically disturbed microbial community before FMT, the increase in alpha diversity after FMT was greater than that of RCPT/B (Wilcoxon test, p < .05), suggesting the efficiency of FMT in enhancing the diversity of the disturbed gut microbiota.

We next analyzed the variation in dominant bacteria in the two enterotypes of recipients and donors. In contrast to alpha diversity, the relative abundance of the dominant bacteria in recipients after FMT decreased toward the level of the donor, especially for RCPT/E (Figure 2b). Moreover, such a decrease in relative abundance was also significant in other enterotype-characteristic genera including *Citrobacter, Enterobacter* and *Acinetobacter* (Wilcoxon test, p < .05) (Supplementary Figure S5b), which illustrated the effect of FMT donors on modulating microbial community structure.

To quantify the donor’s contribution to modulating recipients’ microbial community, we calculated the Bray-Curtis dissimilarity (BC distance) among these samples and performed principal coordinates analysis (PCoA) (Supplementary Figure S5d). Recipients (before and after FMT) and donors formed three distinct clusters along principal coordinate 1 (PC1). After FMT, the recipients were closer to the donors along PC1 than to themselves before FMT (Wilcoxon test, p < .05). We then calculated the BC distance between each recipient and its corresponding donor over time. The distance in both enterotypes decreased during the first few weeks after FMT and reached a minimum at approximately two months (Supplementary Figure S6a), potentially reflecting the engraftment and bloom of donor-derived bacteria.

To explore the underlying links between donor-derived bacteria and FMT efficacy, we further focused on recipients with known FMT outcomes (response/failure). We found that the recipients with different outcomes clearly formed two groups based on the microbial BC distance to their corresponding donors and themselves before FMT (ANOSIM test, statistic = 0.11, p = .001, Figure 2c), regardless enterotype and disease (Figure 2d-f and Supplementary Figure S5f and S6). We further assessed the association between FMT outcome and the distance between recipients after FMT and their donors and found that the distance was shorter in the response group than in the failure group (Wilcoxon test, p < .05) (Supplementary Figure S5e).

We next examined the potential sources of the bacteria that were significantly associated with FMT outcomes. In total, 16 taxa were identified to be enriched or depleted in the response group of recipients after FMT (Wilcoxon test, q < 0.05) (Supplementary Figure S5g). We compared the cumulative abundance of these taxa between the two potential sources (donors and patients before FMT) (Figure 2d-f and Supplementary Figure S6). As expected, the cumulative abundance of enriched taxa was significantly higher in donors and recipients after FMT than in recipients before FMT (Wilcoxon test, p < .05), whereas the opposite trend was found in the depleted taxa. Taken together, these findings strongly indicate that the engraftment of donor-derived bacteria in FMT contributes to symptom alleviation in recipients.

### Bacterial engraftment in recipients

**Figure 3.**
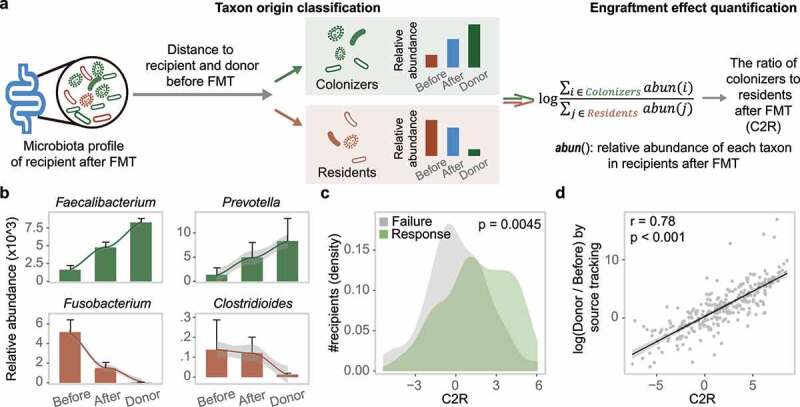
Bacterial engraftment in recipients. (a) Schematic of how to estimate the ratio of colonizers to residents after FMT (C2R). Gut bacteria in recipients after FMT were classified into colonizers and residents based on distance to donors and themselves before FMT. The C2R was calculated by the ratio of summed abundance between colonizers and residents in log-space. (b) Mean relative abundance during FMT of *Faecalibacterium, Fusobacterium* in all patients, *Prevotella* in IBD patients, and *Clostridioides* in CDI patients. The error bar indicates the 95% confidence interval. (c) C2R in the response and failure groups (n = 286, Wilcoxon test, p = .0045). The density of the number of recipients was depicted in the y-axis. (d) C2R and similar equations obtained by source tracking (Spearman correlation, r = 0.78).

To validate the contribution of the engraftment of donor-derived bacteria to FMT success, we sought to quantify the proportion of bacteria that may be derived from the donors. We first classified the gut bacteria in recipients after FMT into two categories, residents and colonizers (Figure 3a). We assumed that residents were abundant in patients before FMT, whereas colonizers were absent or low in abundance in patients before FMT but newly acquired from donors. Each taxon was assigned to one category based on the change in its relative abundance during FMT by comparing the distance of the taxon’s abundance after FMT to that of the donor and the patient before FMT. We then designated the ratio of colonizers to residents after FMT (C2R) by dividing the summed abundance of colonizers by that of residents. A higher C2R indicates that more bacteria from the donor have successfully colonized the recipient’s gut. Since colonizers and residents can be clearly distinguished by this method, the C2R provides a comparable and traceable measurement for microbiota analysis in FMT studies.

Among the 20 most abundant gut bacteria in the 286 recipients after FMT, 5 and 15 of them could be classified as residents and colonizers, respectively. The relative abundance of the 15 colonizers accounted for 60.2% of the total, implying intense colonization by donor-derived microbes in recipients. *Fusobacterium* and *Streptococcus* were categorized as residents, as their abundance was closer to that in patients before FMT than to that in donors (Figure 3b and Supplementary Figure S7). Additionally, the two enterotype-dominant taxa (Enterobacteriaceae and *Bacteroides*) were also classified as residents in the respective patients (Figure 2b). In contrast, due to their increased abundance after FMT, reaching the level of donors, *Faecalibacterium* and *Prevotella* were categorized as colonizers (Figure 3b and Supplementary Figure S7).

Using the abundances of all residents and colonizers, we calculated the C2R for each recipient, and found that the C2R was significantly elevated in the response group compared to the failure group (Wilcoxon test, p = .0045) (Figure 3c), which was consistent in both diseases (Supplementary Figure S7e). Generally, the decreased C2R in the failure group was mainly attributed to the retention of residents, especially pathogenic bacteria, during FMT. Eighty-six residents were identified in the failure group, whose relative abundance was twice that of the response group (31.6% vs. 15.8%). For example, the level of *Clostridioides* was significantly higher in the failure group of CDI recipients after FMT (Supplementary Figure S7d), which in turn reduced the C2R. In addition, several taxa in donors were negatively correlated with *Clostridioides* in recipients after FMT (*Lachnospira*, r = −0.17; *Bacteroides*, r = −0.14, Supplementary Table S3), indicating the significance of selecting suitable donors to reduce pathogenic residents in patients.

To further validate the applicability of the C2R, we first performed source tracking analysis using a state-of-the-art method (FEAST^40^) on these microbiota datasets (see Methods). The microbial profiles of donors and patients before FMT were used as potential sources in the input to FEAST, and the fold change was calculated using the probability from the two potential sources to mimic the C2R. As shown in Figure 3d, the distribution of C2R fit well with the results of FEAST, with traceable bacteria and fewer outliers (Spearman correlation, r = 0.78). We then analyzed two shotgun sequencing-based FMT datasets from a CDI study^19^ and an IBD study,^41^ and found that a similar transmission trend was reproduced based on the residents (e.g. *Fusobacterium* and Enterobacteriaceae) and the colonizers (e.g. *Faecalibacterium* and *Prevotella*) in the 16S rRNA datasets (Supplementary Figure S8 and S9). In addition, the C2R was higher in the response group compared to that in the failure group (Supplementary Figures S8e and S9c). Taken together, these findings demonstrated the robustness and scalability of the C2R.

### Enterotype-based donor-recipient matching contributes to FMT success

**Figure 4.**
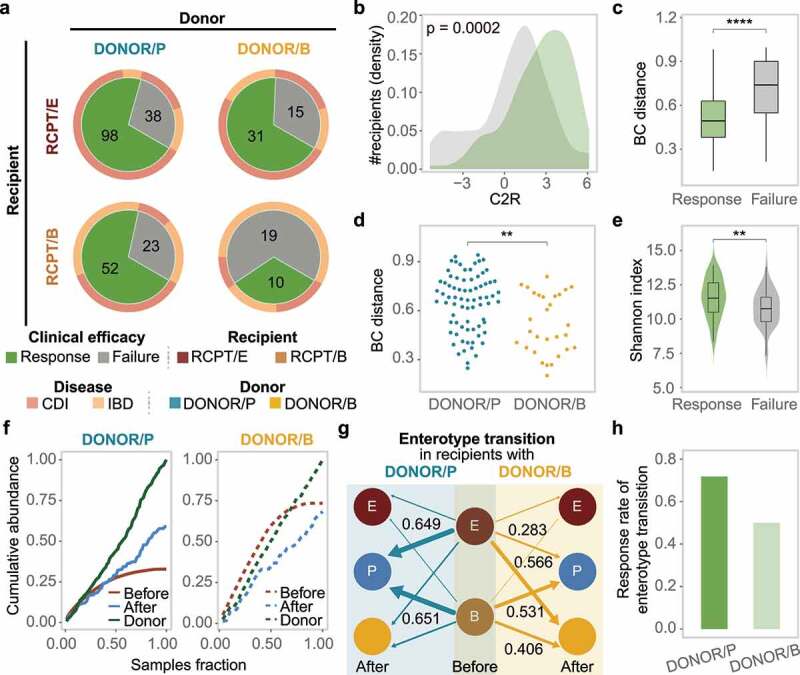
Enterotype-based donor-recipient matching contributes to FMT success. (a) FMT efficacy for different enterotypes of recipients and donors (n = 286). The statistic represents the number of patients who received FMT. (b-c) C2R (b) and Bray-Curtis (BC) distance (c) between the response and failure groups of RCPT/E after FMT (n = 182). The density of the number of recipients was depicted in the y-axis (b). (d) BC distance between RCPT/B patients and donors of DONOR/P or DONOR/B (n = 104). (e) The summed alpha diversity between the response group and the failure group of RCPT/B (n = 104). The summed alpha diversity was calculated by summing the Shannon alpha diversity of paired recipients and donors. (f) The cumulative abundance curve of donor-specific genera in recipients or donors during FMT. Donor-specific genera were those with significant differences in abundance between DONOR/P and DONOR/B (Wilcoxon test, q < 0.05). Cumulative abundance was calculated by summing corresponding donor-specific genera in recipients before or after FMT. To make this measure comparable between the two donor enterotypes, the cumulative abundance was normalized to the level of corresponding donors separately (DONOR/P or DONOR/B). The sample fraction was normalized by the number of recipients in each group (transplanted by DONOR/P or DONOR/B). (g) Enterotype transition in recipients treated with different donors (DONOR/P and DONOR/B). Blue and yellow lines represent DONOR/P and DONOR/B, respectively. The number represents the fraction of enterotype transitions, and only fractions higher than 0.25 are depicted. (h) The response rate of recipients with enterotype transition. DONOR/P and B represent the enterotypes of the donor for the recipients. Asterisks indicate significance (****p < .0001, ***p < .001, **p < .01, *p < .05).

Since the engraftment of donor-derived bacteria was significantly associated with FMT outcome, we wanted to know which enterotype of donors was more effective for CDI or IBD patients. We first clustered the donors’ gut microbiota profiles following the same approach as that used for patients. The donors could be divided into two enterotypes, DONOR/P and DONOR/B, named according to their dominant taxa, *Prevotella* and *Bacteroides*, respectively (Supplementary Figure S10). The two donor enterotypes had similar microbial diversity (Wilcoxon test, p > .1) and age distribution (Wilcoxon test, p > .1) (Supplementary Figure S10). We next measured the response rate for each enterotype of recipients with different donor types. As shown in Figure 4a, RCPT/E exhibited the highest clinical response rate (70.88%), regardless of the donor’s enterotype. As expected, the C2R in RCPT/E was markedly elevated in the response group compared to the failure group (Wilcoxon test, p = .0002), and the BC distance between donors and patients was also reduced after FMT in the response group (Figure 4b and 4c). However, the clinical efficacy in RCPT/B was significantly affected by donor enterotype (chi-square test, p < .005) (Cochran-Mantel-Haenszel test blocked factor “corticosteroids history”, p = .02), in which the response rates under transplant with DONOR/P and DONOR/B were 69.33% and 34.48%, respectively (Figure 4a), which was consistent in both CDI and IBD (Supplementary Figure S11). These findings indicate the potential role of enterotype matching between patients and donors when choosing suitable FMT donors.

To determine why DONOR/P was more effective against RCPT/B than DONOR/B, we first compared the microbial BC distances of RCPT/B to the two donor enterotypes. As shown in Figure 4d, the distance to DONOR/P was significantly greater than that to DONOR/B (Wilcoxon test, p < .05), which was consistent in both diseases (Supplementary Figure S11). In addition, the alpha diversity of donors and recipients was significantly higher in the response group than in the failure group especially in CDI and UC (Wilcoxon test, p < .05) (Figure 4e and Supplementary Figure S11), indicating that the recipients may have a higher possibility of engrafting more diverse bacteria from more distantly related donors.

To illustrate the engraftment superiority of distantly related donors in RCPT/B, we obtained the bacteria enriched in each of the two donor enterotypes (Wilcoxon test, q < 0.05) and calculated the cumulative abundance of these bacteria. As shown in Figure 4f, the cumulative abundance in recipients treated with DONOR/P was doubled after FMT, but it was reduced in recipients treated with DONOR/B, indicating more diverse donor-derived microbes were transplanted with DONOR/P compared to DONOR/B. It should be noted that the original cumulative abundance in recipients before FMT was much higher in the DONOR/B group than in the DONOR/P group. We next focused on the fraction of the enterotype transition during FMT in all recipients, which measured the percentage of patients whose enterotype was changed to their donors’ enterotype (see Methods). As expected, the fraction of enterotype transition was high for both RCPT/E (62.7%) and RCPT/B (58.5%). Notably, in RCPT/B recipients, enterotype transition was more frequent when treated with DONOR/P (65.1%) than when treated with DONOR/B (40.6%), confirming the engraftment superiority of DONOR/P. Among these recipients with enterotype transition, we found that the response rate was also higher in DONOR/P-treated recipients than in DONOR/B-treated recipients (76.7% vs. 50%). These findings demonstrated the efficacy of distantly related donors in reshaping the recipients’ gut microbiota and improving the FMT response rate.

### Construction and validation of the enterotype-based donor selection model

**Figure 5.**
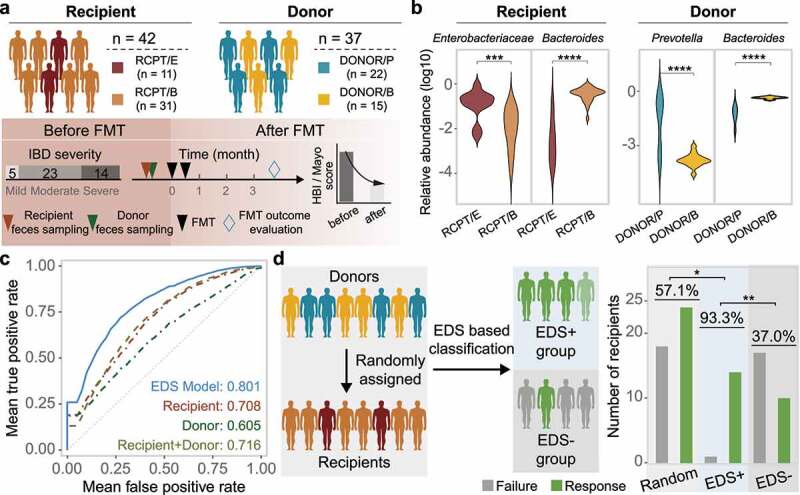
Construction and validation of the enterotype-based donor selection model. (a) Outline of the validation cohort (n = 42). Characteristics of recipient and donor are depicted in the upper and bottom panel. Response rate was determined based on a at least 3-month follow-up for each recipient. (b) Dominant taxa of recipients and donors from different enterotype in the validation cohort. Enterotype of each individual was assigned by the medoids of the known enterotypes. *Enterobacteriaceae* represents the dominant genus in RCPT/E. (c) Receiver-operating characteristic (ROC) curves for the enterotype-based donor selection (EDS) model and other alternative models by 5-fold cross-validation with 500 iterations in CDI and IBD (n = 286). (d) The performance of the EDS model in the validation cohort (n = 42). The left panel depicts the schema of donor assignment (the random group) and by the EDS model (the EDS group). The right panel depicts the distribution of FMT outcomes (response and failure) in the random group and the EDS group. Asterisks indicate significance (****p < .0001, ***p < .001, **p < .01, *p < .05).

To evaluate the application of enterotype-based donor-recipient matching in FMT, we built a machine learning model to match patients with suitable donors, and validated it using both cross-validation and an additional FMT cohort with 42 newly recruited IBD patients (Figure 5a and 5b). A random forest model leveraging enterotype was trained to predict the outcome of FMT for each recipient-donor pair (Supplementary Figure S12). The input features of the enterotype-based donor selection (EDS) model included enterotypes of recipients and donors before FMT and their corresponding microbial profiles (Methods and Supplementary Table S4). We calculated the average area under the ROC curve (AUROC) of the EDS model in all patients analyzed above (n = 286) by 5-fold cross-validation with 500 iterations (Figure 5c); the AUROC was 0.80, which showed high specificity and sensitivity in FMT outcome prediction.

We further compared the EDS model against three alternative models, in which the input features included microbial profiles from only patients, only donors, or both. Consistent with the EDS model, random forest model was also used for prediction in the three alternative models with same parameters. Through 500 iterations of 5-fold cross-validation, we found that the EDS model outperformed all three alternative models (Wilcoxon test, p < .05) (Figure 5c), indicating that enterotype-based donor-recipient matching can improve model performance in FMT outcome prediction.

Although model performance was evaluated using cross-validation, we sought to validate it by recruiting an additional validation cohort of IBD patients (n = 42, Figure 5a and Supplementary Table S5). Characteristics of this validation cohort were similar to the previous discovery cohort, including gender, age and disease severity. We assigned enterotype for each individual in this new cohort by calculating its microbial distance to the medoid of known enterotypes (Supplementary Figure S12a). As expected, the dominant taxa of these individuals were consistent with their enterotype classification (Figure 5b). We applied the EDS model to match patients with donors and selected highly promising patient-donor pairs based on the assigned enterotype in the validation cohort, which formed the EDS+ group (n = 15) after selection. The remaining patients which were not selected by the EDS model in the validation cohort formed the EDS– group (n = 27). The response rate was 93.3% in the EDS+ group, which was much higher than that in the validation cohort with randomly assigned donors (57.1%, n = 42) (Odds ratio, 10.5 [95% CI, 1.26–87.37]; Chi-square test, p < .05) and the EDS– group (37.0%, n = 27) (Odds ratio, 23.8 [95% CI, 2.7–209.3]; Chi-square test, p < .01) (Figure 5d). In addition, the BC distance between the donors and RCPT/B in the EDS+ group was also significantly higher than that in the EDS- group (Supplementary Figure S12). Taken together, these findings suggest that the EDS model may be useful for selecting suitable FMT donors for CDI or IBD patients in clinical practice.

## Discussion

By restructuring the recipient gut microbiota with donor fecal samples, FMT has been used to treat many diseases, such as infections^11^ and metabolic disorders.^10^ However, how to choose a suitable donor for patients has not been fully elucidated. In this study, we applied a conceptual framework to the human gut microbiota to obtain more insights into donor-recipient matching and thus built an enterotype-based model for donor selection. By combining our two recruited cohorts treated with FMT and additional 14 public FMT cohorts, we identified the existence of two robust enterotypes (RCPT/E and RCPT/B) in both CDI and IBD patients. We demonstrated that the engraftment of donor-derived microbes was significantly associated with FMT outcome and discovered that DONOR/P was more suitable for treating RCPT/E patients. By leveraging enterotype-based classification of the gut microbiota in donors and recipients, we proposed an EDS model for selecting suitable donors for patients, which was confirmed through statistically rigorous cross-validation and an independent IBD cohort, achieving a response rate of 93.3%.

Although CDI and IBD are two distinct entities, they are overlapped in many ways, for example symptoms and associated with gut microbiome dysbiosis. Most importantly, both diseases are recurrence to convention therapy, but could be responsive to FMT. Studying this common phenomenon is a novel aspect of our study. In previous studies,^42–44^ researchers focused on one disease and neglected the commonalities in the microbial interactions of FMT. In this study, we introduced enterotype to find the microbial similarity between the two diseases. In the light of enterotype, patients of the two diseases can be divided into two conservative enterotypes (RCPT/E and RCPT/B), which exhibit similar microbial compositions (Figure 1). The two enterotypes were pervasive in both CDI and IBD patients with a separate preference (Supplementary Figure S3c), suggesting enterotype is a reliable criterion to differentiating gut microbial characteristics of patients with the two diseases. In addition, we found strong enterotype matching in FMT efficacy (Figure 4a), which can be used to interpret the commonalities of this treatment.

In this study, we found that both alpha diversity and BC distance in recipients’ gut microbiota resemble those of the donors’ after FMT. In a previous metagenomic analysis of 19 CDI patients, nearly 40% of the strains from donors were found in post-FMT patients, and the abundances of the strains were significantly enhanced.^19^ Similarly, in another study of the Human Microbiome Project (HMP) cohort, transplanted microbes were found to engraft and colonize the patients’ gut for months,^45^ which is in line with our finding on the reduced BC distance between recipients and donors over time, implying the engraftment and bloom of the donor-derived microbiota in the recipients’ intestines. Engraftment of the donor-derived microbiota in recipients may help improve patients’ gut microecology and health. For example, a significant increase in short-chain fatty acid-producing microbes, particularly *Eubacterium*, was observed in FMT-treated CDI patients.^46,47^ The engraftment of some species in *Actinobacteria*, was found to be linked with better FMT outcomes in IBD patients.^41^ However, because of restrictions in sample size and analytical methods, the quantitative correlation between the engraftment of donor-derived bacteria and FMT outcomes has not been clearly elucidated. In this study, we illustrated a significant association between donor-derived bacterial engraftment and FMT outcomes by introducing a new quantitative measurement: the ratio of colonizers to residents after FMT (C2R). Our findings demonstrate that the engraftment of donor-derived microbes in patients after FMT can be used as an indicator for FMT success.

Currently, FMT donor screening criteria mainly relate to biosafety issues to ensure that donors do not pose any risk of transmissible adverse factors, such as pathogens or diseases.^48^ Donors selected according to these criteria may have a healthy gut microbiome, but it is unlikely to be suitable for all patients with different clinical and microbiota backgrounds.^16^ For example, previous studies found that the colonization and therapeutic effect of a transplanted microbiota from the same donor differed greatly among patients.^9,49^ One solution is to find beneficial microbes for disease curation, such as *Akkermansia* for IBD,^50^ which is transplanted into patients with a designed beneficial microbiota formulation. However, the outcomes of this strategy in CDI trials were vague,^51,52^ probably because certain beneficial bacteria cannot survive or fulfill their function without a complete microbial community. In this study, we proposed a new EDS model for evaluating donor-recipient matching and improving FMT efficacy mainly for moderate and severe patients in our cohort. The two enterotypes (RCPT/E and RCPT/B) used in the EDS model have been confirmed by several recent studies. In a study of 57 patients who suffered from CDI, for example, gut microbiota samples were clustered into two enterotypes, with Bacteroidaceae and Enterobacteriaceae being the two most abundant taxa.^42^ Similarly, in the Meta-HIT cohort^43^ and HMP cohort,^44^ the enterotype *Bacteroides* was found in the majority of IBD patients, whereas Enterobacteriaceae was predominant in the other IBD patients. Considering that FMT is a therapy used to restructure the recipient’s gut microbial community through bacterial transmission and engraftment, recipients with similar community structures may be good matches for the same kind of donor. Our study found that patients with different enterotypes (RCPT/E and RCPT/B) responded best to different donor selection guidelines. For RCPT/E patients, there was little difference in response rate when selecting donors with different enterotypes, particularly in CDI. For RCPT/B patients, donors dominated by *Bacteroides* were not suitable for transplantation. However, distantly related donors, such as DONOR/P, were more effective, which showed a high possibility of engrafting more diverse bacteria. A meta-analysis of 319 patients with IBD showed that receiving fecal bacteria from a close related donor (e.g., a family member or close friend) was less effective than from a distant donor, with a 16% decrease in clinical remission.^53^ And certain species in the dominant taxon in DONOR/B (e.g. *Bacteroides*) were associated with poor outcomes in human clinical studies in IBD patients,^41^ further suggesting that DONOR/P may be an optimal option for treating IBD patients with RCPT/B.

This study still has several limitations. First, our work mainly focused on 16S rRNA amplicon sequencing data, which is limited in the resolution of taxonomic assignment and function inference. Metagenomic sequencing can provide enough phylogenetic resolution to strain level, which would be great helpful for engraftment estimation.^54^ By analyzing two metagenomic datasets (CDI and IBD) (Supplementary Figures S8 and S9), we found that two enterotypes (RCPT/E and RCPT/B) were prevalent in patients with IBD or CDI. And our proposed C2R was scalable and robust in the metagenomic datasets, where colonizers and residents were consistent at a higher resolution. Second, our meta-analysis was limited by the inability to adequately control for the factors that may potentially influence gut microbiome and FMT efficacy, including biological factors (fungi,^33^ virus^29^), dietary factors^55^ and medicines.^48^ Future clinical studies with comprehensive design to measure the effect of these factors would contribute to the standardization of FMT.

## Conclusions

Our study reveals the consistency of two enterotypes between CDI and IBD, and based on enterotype matching between recipients and donors, we propose an EDS model to improve FMT outcomes. This study also provides a quantitative measurement to bridge the engraftment of donor-derived microbes and FMT outcomes, which may be the foundation for precision FMT. With the accumulation of FMT multi-omics datasets, we believe that more robust machine learning-based methods will be developed to improve the clinical treatment of intestinal diseases.

## Methods

### IBD cohort recruitment and FMT procedure

We enrolled a cohort of IBD patients treated with FMT. This clinical trial was registered as NCT01793831 and NCT01790061, and approved by the Second Affiliated Hospital of the Nanjing Medical University Institutional Ethical Review Board. A part of this clinical trial has been published previously.^21,56^

Patients with IBD were recruited based on our inclusion criteria (see details in Supplementary Methods). All patients who had poor response to convention therapy, for example, 5-aminosalicylic acid, corticosteroids, immunomodulators, and willing to receive FMT were included. Corticosteroids were required to taper off at least 1 week before FMT, and other medications were stopped except 5-aminosalicylic acid prior to the first FMT. Probiotics or antibiotics were not suggested after FMT. The baseline characteristics of eligible participants were recorded, including age, gender, diagnosis, Mayo score/Harvey–Bradshaw index, and relationship with the corresponding donor (Supplementary Table S1). All subjects underwent laboratory examinations (routine blood examination, C-reactive protein, erythrocyte sedimentation rate, hepatorenal function, etc.) before FMT. Endoscopy and total abnormal magnetic resonance imaging were used to assess the condition of the patients when necessary. Due to histology or endoscopy, which can accurately reflect disease severity, was not practical for each patient, Mayo score/Harvey–Bradshaw index was used to evaluate disease activity.

The donors mainly came from Chinese fmtBank (China Microbiota Transplantation System) and patients’ relatives or friends who met our strict criteria (see Supplementary Methods).^57^ The FMT procedure, involving the preparation of washed microbiota suspension,^58^ the step-up FMT strategy and FMT delivery methods, was the same as the procedure published previously.^21,56^ 3–5 Units of fresh fecal bacteria (1 U = 1 × 10^13^ cells) in suspension (1 U with 20 mL saline) according to patients’ conditions^57,58^ was delivered through one of the three delivery ways: endoscopic, nasojejunal tube or transendoscopic enteral tubing.^56^ The decision on the delivery method was made based on the patient’s characteristics and medical application conditions. Prior to transplantation, no antibiotics or/and colon washing were specifically ordered for bowel preparation. However, it is not practical to exclude the following patients with potential bowel preparation: the patients with previous antibiotic treatment for intestinal infections or infections beyond gut, or patients underwent colonic endoscopy for evaluation. Clinical response was evaluated based on the criteria in the Supplementary Methods at 3 months after FMT or the last medical visit (≥ 3 months).

Stool samples were collected from patients and donors in sterilized Eppendorf tubes and stored at −80°C.^59^ These samples were used to extract microbial DNA for sequencing. The V4–V5 region of the bacterial 16S rRNA gene was amplified using Phusion High-Fidelity PCR Master Mix with HF buffer (New England Biolabs, England) and then sequenced on the Illumina MiSeq platform (Illumina, Inc.) using standard Illumina sequencing protocols.^60,61^ Only patients with complete pre- and post-transplant samples and donor samples were included in downstream analysis.

### Public FMT cohorts used in this study

We used PubMed and the NCBI BioProject to search for studies that included fecal metagenomic sequence data of human FMT patients and donors (see details in Supplementary Figure S1 and Methods). To guarantee the robustness of our meta-analysis,^62^ we manually excluded studies with fewer than 20 human fecal samples, missing sequence data, and unclear metadata (e.g., patient and corresponding donor labels). Most metadata were downloaded from the NCBI SRA database or supplementary files provided in the original publications, but some were acquired after communicating with the authors by e-mail. To maintain uniformity,^63^ we focused on only the 16S rRNA gene amplicon sequencing data of CDI and IBD patients, which included most studies and samples in the searched hits. Fourteen suitable studies were found, and their corresponding characteristics are listed in Supplementary Table S2. These cohorts in these studies were used as discovery cohorts in downstream analysis.

### 16S rRNA gene amplicon sequence processing

Sequence data were downloaded from the SRA database using the accessions listed in Supplementary Table S2. When paired-end reads were available, they were merged using FLASH with default parameters.^64^ Only merged reads were used in downstream analyses. After that, the software AfterQC was used to perform quality control with default parameters.^65^ To ensure consistency with previous studies, operational taxonomic units (OTUs) were selected against the 13–8 Green Genes release and clustered at a 97% identity using QIIME pick_otu_closed_ref with the following parameters: -strand both, -id 0.97.^66^ Samples with fewer than 2500 OTU counts were removed. We then collapsed OTUs to the genus level with QIIME summarize and adopted standard library size normalization in log-space. All statistical analyses were performed on these normalized genus-level relative abundance data.

### Batch effect analysis

To evaluate the effect of potential confounders related to different FMT cohorts, we applied an ANOVA-type analysis and compared the amounts of mean variance explained for each genus. The explanations were compared across these factors, including FMT status and batch effect (study). Variance estimates were obtained for ranks to account for the non-Gaussian distribution of the microbiota abundance data. To further eliminate the potential batch effect, we also employed a blocked version of the Wilcoxon test in the coin package in R by treating ‘study’ as a blocking factor in all analyses unless otherwise specified.^67^

### Enterotype analysis

We first clustered the gut microbiota of the patients by following a previously published tutorial.^68^ To decrease noise, a genus was discarded if its average abundance across all samples was below 1%. Samples were clustered by partitioning around medoids (PAM), and the optimal number of clusters was estimated using the Calinski-Harabasz index. Samples were projected into two dimensions and visualized through principal coordinates analysis (PCoA) by the “dudi.pco” function in the ade4 package in R. Cluster dissimilarity was measured by the “adonis” function in the vegan package in R. The dominant taxon in each enterotype was identified based on the significance level, fold change and relative abundance between enterotypes. Enterobacteriaceae was identified as the dominant taxon in the RCPT/E, because it was abundant and significantly enriched in this cluster (Wilcoxon test, q < 0.001). Four of the top five differential genera in the RCPT/E (Wilcoxon test, q < 0.001) were from Enterobacteriaceae. To make the dominant taxon comparable between RCPT/E and RCPT/B, the most abundant and significantly differential genus in Enterobacteriaceae (Wilcoxon test, q < 0.001) was used to represent the dominant genus in the subsequent analyses.

The consistency of the two enterotypes was assessed by three approaches: the PAM separate approach, PAM combined approach,^68^ and Dirichlet multinomial mixtures (DMM) combined approach.^36^ In the PAM separate approach, samples from patients with the two diseases (CDI and IBD) were clustered using PAM separately. In the combined approach, all samples from patients with the two diseases were mixed and clustered by PAM and DMM. Clustering results were obtained with the three approaches, and consistency was measured as the number of common individuals in the same clusters and common marker genera afterward.

### Microbiota diversity and enrichment analysis

To analyze the dynamics of the patient microbiota, we measured the alpha and beta diversities of the patients and the donors. Alpha diversity was calculated based on the relative abundance of non-collapsed OTUs by QIIME alpha_diversity with the Shannon metric.^66^ Bray-Curtis distance (BC distance) was calculated by vegan in R with the genus abundance. Principal coordinates analysis (PCoA) of the pairwise BC distance matrix of all samples was performed using the ade4 package in R. We also compared the variation in BC distance between the patient and the corresponding donor over time, and the trend was fitted with the “ggline” function in R. We also narrowed down the patients to those with clear FMT outcomes and compared their BC distances between the two enterotypes. Corresponding FMT outcomes were assessed according to original studies and criteria listed in the Supplementary Methods. The cumulative abundance of response-enriched or response-depleted was calculated based on the sum of significant bacteria with FMT outcomes (Wilcoxon test, q < 0.05).

### The ratio of colonizers to residents after FMT (C2R)

To quantify the ratio of residents and colonizers in the patients after FMT, we calculated the C2R for each patient. We first calculated the distance (log fold change) of each taxon between the recipient after FMT and the patient or donor before FMT. Two categories, residents and colonizers, were distinguished based on the distance. Residents were classified as having a shorter distance between the recipients before FMT and after FMT. Colonizers were classified as having a shorter distance between the recipients after FMT and the donors before FMT. We then summed the abundance of all taxa in the two categories separately. Finally, the ratio of the summed abundance of the two categories was defined as the C2R. FEAST was used as a source tracking tool to validate our C2R with default parameters.^40^ The microbiota profiles of donors and recipients before FMT were used as the sources, and the microbiota profiles of recipients after FMT were used as corresponding sinks.

### Enterotype matching and engraftment potential analysis

To explore the enterotype matching between recipients and donors in FMT, the enterotype clustering of donors was analyzed as that performed in the recipients (Supplementary Figure S10a-c).^68^ DONOR/P and DONOR/B denoted the two enterotypes identified in the donors, respectively. To compare the engraftment of donor-derived taxa, we first filtered out donor-specific taxa which were significantly differed between the two enterotypes (DONOR/P and DONOR/B) (Wilcoxon test, q < 0.05). And then cumulative abundance was calculated in the recipients before and after FMT by summing up the abundance of these differential taxa. To eliminate the difference in sample size, we used the sample fraction (from 0 to 1) and depicts cumulative abundance against it in Figure 4f. To compare the engraftment potential between the two donor enterotypes and eliminate the noise from persistent bacteria, the enterotype transition was measured by whether a patient’s enterotype changed to the donor’s enterotype after FMT. The enterotype of recipients after FMT was measured by the distance to the medoids of the three enterotypes identified above, and the enterotype of the nearest medoids was assigned (Supplementary Figure S12).

### FMT outcome modeling

To predict the FMT outcomes for each patient before FMT, we built an enterotype-based donor selection (EDS) model by the random forest package in R. The input features of the enterotype-based donor selection (EDS) model included enterotypes of recipients and donors before FMT and their corresponding microbial profiles, like microbial diversity and abundance of certain taxa (mean abundance > 1%). In the EDS model, random forest classifiers were trained to select features and predict FMT outcomes for the two enterotypes separately. We used the predicted value from the EDS model to evaluate the degree of matching between patients and donors. For a new patient waiting for a suitable FMT donor, we first distinguished their enterotype based on the distance to two enterotypes’ medoids and applied the EDS model to predict FMT outcomes for each potential donor (Supplementary Figure S12).

To compare the performance of the EDS model with three alternative models without the enterotype feature, we shuffled the discovery cohorts by 5-fold cross-validation with 500 iterations. The alternative models used the same machine learning classifier (random forest) as the EDS model. The input features of the alternative models included only the microbial profiles from patients, donors, or both. We measured the mean area under the ROC curve (AUROC) of each model based on 500 iterations of 5-fold cross-validation using the ROCR package in R.

To validate performance, we used the EDS model trained on the discovery cohorts analyzed above for selection in the validation cohort, which formed the EDS selection group. The matching degree of each patient-donor pair in the EDS group was predicted by the EDS model. A higher matching degree means a higher probability that the patient-donor pair would achieve a response after transplantation. The optimal threshold of the matching degree was determined based on the mean of Youden index and the point closest to the top-left part of the ROC curve.

### Statistical analyses

To evaluate the sample size in our analysis, two calculation formula (“pwr.t.test” and “pwr.chisq.test” function) from the pwr package in R were applied. At 80% power and 5% significance level, averaged sample size is 121 per group with mean values and standard deviation of the response and failure groups at the genus-level. For the key conclusion “DONOR/P is more suitable for RCPT/B than DONOR/B in FMT”, the power was at 0.90 (CDI and IBD patients combined) and 0.80 (IBD patients only) with 5% significance level and “pwr.chisq.test” function.

Unless otherwise stated, statistical analyses were performed and figures were created in R (version 3.6) with the ggplot2 package. We performed univariate analyses on a blocked version of the Wilcoxon test with the coin package in R. Wilcoxon test in the main text represents Wilcoxon rank-sum test (Mann–Whitney U test). All p values resulting from multiple hypothesis testing in all analyses were adjusted with the Benjamini-Hochberg false discovery rate using the p-value package in R. The adjusted p value is referred to as the q value in the main text. Descriptions of sample size are available in the main text and accompanying figure legends, where n typically reflects the number of patients. Individual data points are shown where possible, and the error bars in the bar plots represent as the 95% confidence interval of the mean.

## Supplementary Material

Supplemental Material

## Data Availability

Raw sequencing data have been deposited in the BIDG with the accession number PRJCA006255. Code for data analysis is available at https://github.com/bioinfo-biols/FMT_Enterotype.
