# Supplementary material for: The interplay of gut microbiota between donors and recipients determines the efficacy of fecal microbiota transplantation
Source: Gut Microbes. 2022 Jul 19;14(1):2100197. doi: 10.1080/19490976.2022.2100197 (PMC9302524; doi:10.1080/19490976.2022.2100197)
Supplement: Supplemental Material [file KGMI_A_2100197_SM8864.pdf]

Supplementary Methods

Patients recruit criteria

Patients inclusion criteria were: (1) patients definitely diagnosed with inflammatory bowel disease (IBD) on the basis of typical clinical, endoscopic, and histological criteria; (2) active ulcerative colitis (UC) patients with Mayo score ≥ 6 and active Crohn's disease (CD) patients with HBI score > 4 who had poor response to 5-aminosalicylic acid, corticosteroids, immunomodulators, and/or anti-tumor necrosis factor agents; (3) patients provided written informed consents and underwent at least one course of FMT; and (4) patients have been followed up for more than three months, with complete collection of feces samples. Participants were excluded if they accompanied other intestinal diseases, e.g., *Clostridium difficile* infection, severe diseases including malignant cancers, serious liver and kidney disease, cardiopulmonary failure and etc. Corticosteroids were required to taper off at least 1 week before FMT, and other medications were stopped except 5-aminosalicylic acid prior to the first FMT. Probiotics or antibiotics were not suggested after FMT.

Donor screening

The criteria for FMT donor screening include eight dimensions: age, physiology, pathology, psychology, veracity, time, living environment and recipients. We briefly described it as follows.

Inclusion criteria:

(1) age: 6 to 24 years old.

(2) Physiologic criteria

The body growth, body mass index of donors should be within normal value ranges. They have good sleep quality, with normal daily habits, diet, physical exercise and regular bowel habits.

Exclusion criteria:

(1) Pathological criteria

Antibiotic usage within three months, history of asymptomatic/suspected or specific pathogen infection, previous abnormal bowel habits, tattoos or body piercing, allergies, immunological abnormalities, illicit drug usage, incarceration or history of incarceration, high-risk sexual behavior, confirmed genetic risk factors, family members with cancer, diabetes, togavirus, hepatitis viruses, Epstein-Barr virus, cytomegalovirus, human immunodeficiency virus, IBD and immune system diseases, bacterial infections, or other infectious diseases.

(2) Psychological criteria

A history of psychiatric disorders, a definite or suspected psychological disorder and behavior abnormality.

(3) Veracity

Persons judged to have questionable veracity.

(4) Living environment

Living in geographic extremes (regions of high altitude, high temperature, alpine, cold, high humidity, severely polluted areas, and saline-alkaline areas); exposure to epidemic area within the past 3 months.

Public studies inclusion criteria

Our study inclusion and exclusion pipeline have been added to Supplementary Figure S1, and copied as below. In brief, our procedure was composed of three steps:

Firstly, we collected FMT studies from keyword searches in NCBI Bio-project and by following references in related studies. Our search terms are "((((fecal microbiota transplantation) AND (sequencing OR sequence OR metagenome))) AND human)" and "((((((((((Fecal microbial transplant) OR (Fecal microbiota transplantation) OR (FMT)))) NOT chicken) NOT mice)) NOT pig) NOT mouse)) NOT (Canine)".

Secondly, we manually checked these entries and collected them for the next step. Our criteria are: 1) datasets are freely accessible for download; 2) exclude any studies which required additional ethics committee approvals or authorizations for access; 3) exclude studies without CDI or IBD patients; 4) excluded studies with fewer than 20 samples.

Thirdly, we manually excluded studies that are not capable of performing further analyses: 1) exclude studies without 16S rRNA sequencing samples; 2) exclude studies without samples of patient and paired donor before FMT; 3) exclude studies without metadata about which donor was transplanted to the patient; 4) include studies that best contain samples of patient after FMT; 5) include studies that best contain metadata about FMT efficacy. For some studies without key metadata, we have tried to acquire by personal email with the authors.

Outcome assessment of FMT

In our recruited IBD cohorts, efficacy of FMT was evaluated three months after treatment. The clinical response was defined as follows: (1) For UC patients, Partial Mayo score (except endoscopic scores) has a decrease of $\geq$ 2 points and $\geq$ 30% from baseline plus the rectal bleeding sub-score $\leq$ 1 point or the decrease of it $\geq$ 1; (2) The HBI score of CD patients decreased by > 3 points from baseline. Failure to meet the above criteria, or conversion of treatment (e.g. biologics therapy, surgery) were considered non-response.

For the public CDI study, the FMT response was defined as an absence of diarrhea or persistent diarrhea that could be explained by other causes with a negative stool test for C. difficile test in the follow-up after FMT. And for the public IBD study, the FMT response definition was the same as above based on the severity score provided in the paper.

Supplementary Figures

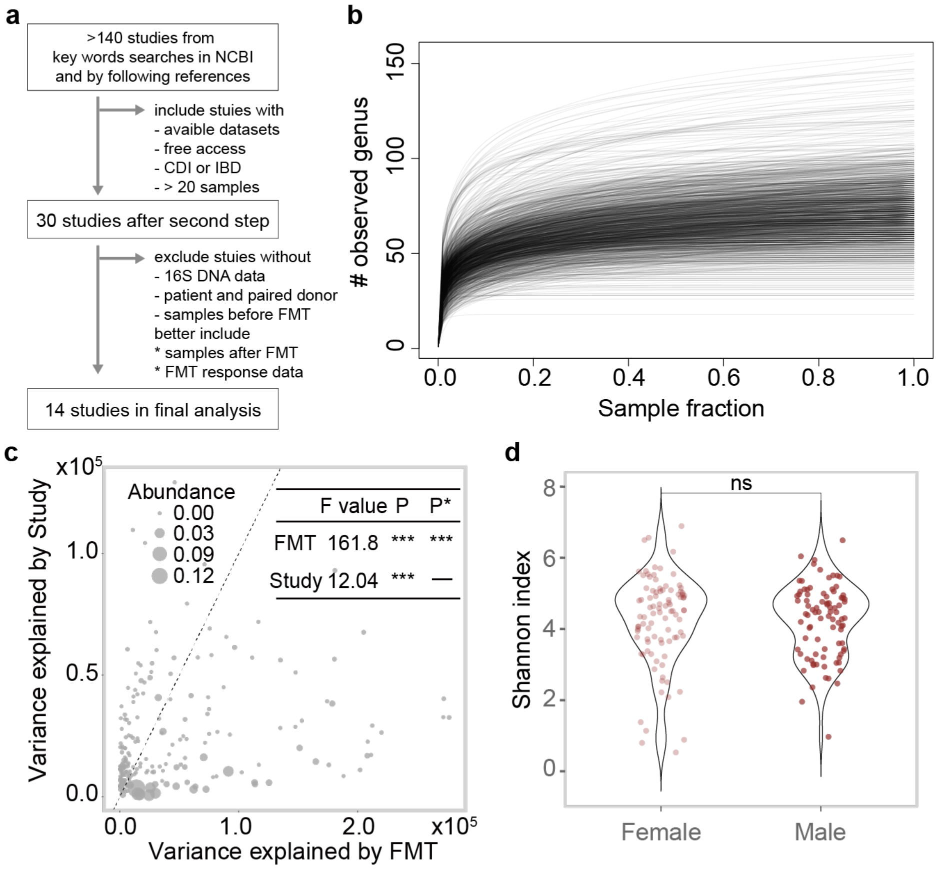

Supplementary Figure S1. Estimation of the effect of confounders compared to that of FMT. (a) Schema of studies inclusion and exclusions. (b) Rarefaction curves of included samples (#sample = 1,440). (b) Mean-variance explanation between two confounding factors ("FMT" and "Study") (n = 322). Dash line represents the equivalence of the two factors. (c) Microbial alpha diversity (Shannon index) of included patients with sex label (n = 181). The "ns" represents p > 0.2 in the Wilcoxon test.

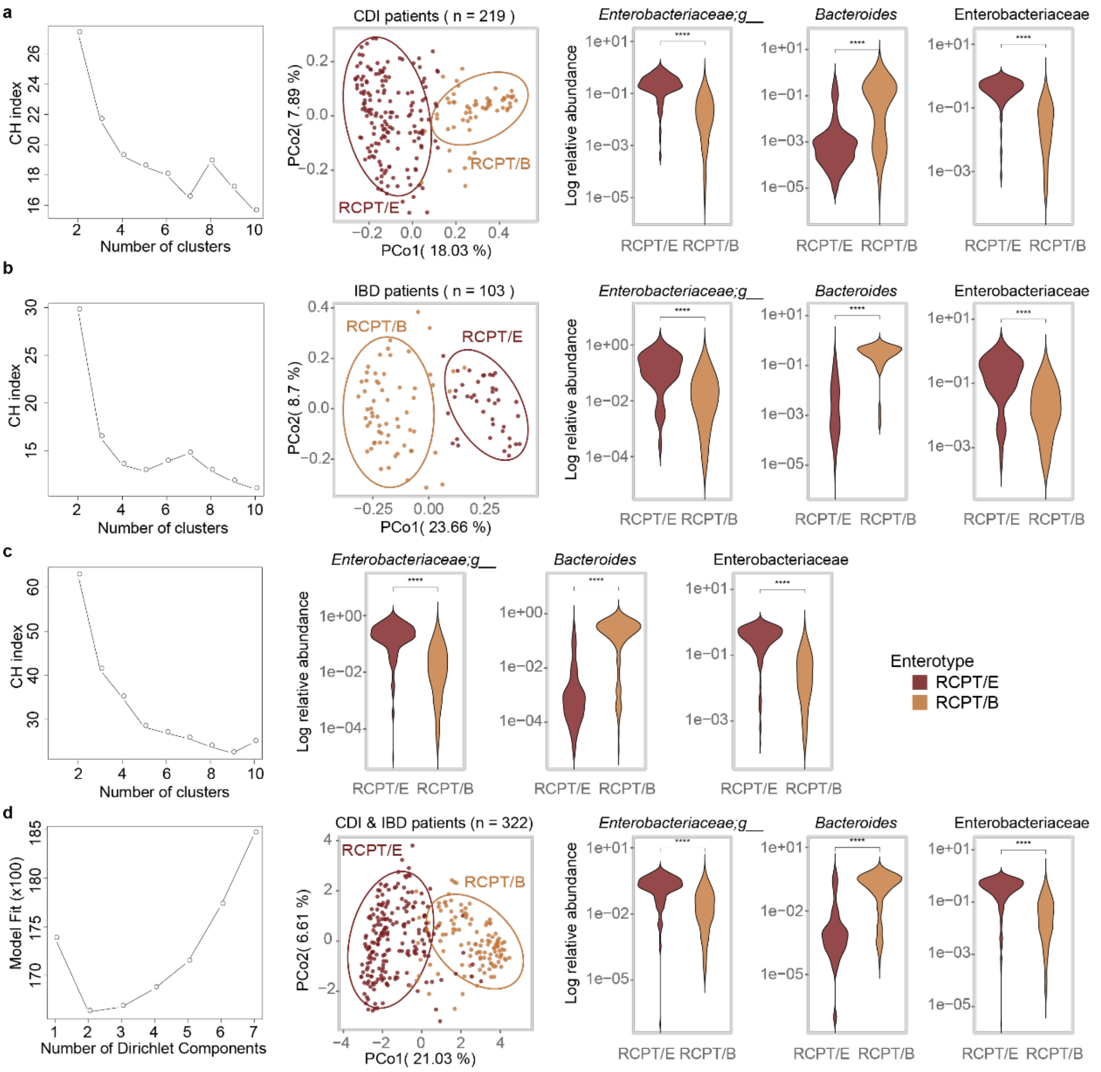

Supplementary Figure S2. Enterotype clustering and dominant taxa of each enterotype. (a-b) Left to right, the optimal number of clusters by CH index, principal coordinates analysis based on Bray-Curtis dissimilarities, and relative abundance of three dominant taxa in CDI (a) and IBD (b). Asterisks indicate significance (****p = < 0.0001, ***p = < 0.001). (c) Left to right, the optimal number of clusters by CH index and relative abundance of three dominant taxa with combined datasets of CDI and IBD by partitioning around medoids. (d) Left to right, the optimal number of clusters by model fit, principal coordinates analysis based on Bray-Curtis dissimilarities, and relative abundance of three dominant taxa with combined datasets of CDI and IBD by Dirichlet multinomial mixtures. Asterisks indicate significance (****p = < 0.0001, *** p = < 0.001).

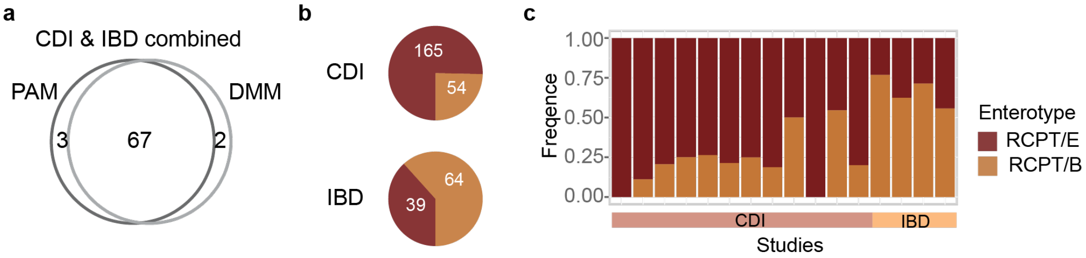

d Smoke

	0	1
RCPT/E	24	3
RCPT/B	26	8

Chi-square test: p = 1
1 represents true

e BMI

	<18	18<
RCPT/E	5	15
RCPT/B	5	16

Chi-square test: p = 1
No patient BMI > 25

f 5-ASA

	0	1
RCPT/E	1	26
RCPT/B	3	31

Chi-square test: p = 0.77
1 represents true

g Immunomodulators

	0	1
RCPT/E	17	10
RCPT/B	22	12

Chi-square test: p = 1
1 represents true

h Corticosteroids

	0	1
RCPT/E	11	16
RCPT/B	4	30

Chi-square test: p = 1
1 represents true

i Severity degree

	1	2	3
RCPT/E	3	16	8
RCPT/B	4	17	13

Chi-square test: p = 0.75

Supplementary Figure S3. Consistency of the two enterotypes (RCPT/E and RCPT/B) by different approaches. (a) Common enterotype-associated genera (Wilcoxon test, $q < 0.0001$) by two approaches (partitioning around medoids and Dirichlet multinomial mixtures) in all datasets. The q value represents the p value adjusted by the Benjamini-Hochberg false discovery rate. (b) Enterotype composition between CDI and IBD. (c) Enterotype composition in each study. (d-i) Associations between enterotype composition and clinical factors in IBD patients, including "smoking tory" (d), "BMI" (e), "5-ASA history" (f), "immunomodulators history" (g) "corticosteroids history" (h), "disease severity degree" (i).

a

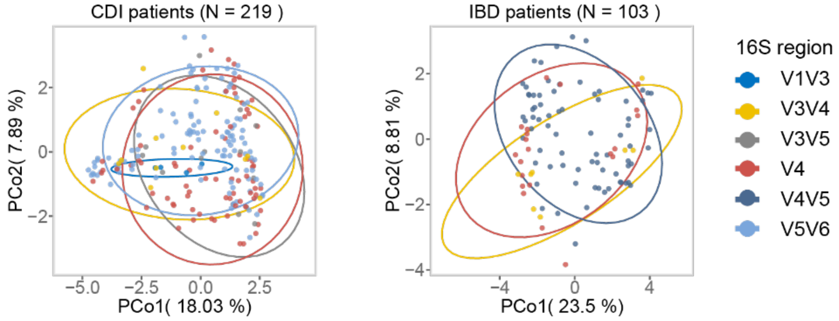

b

Sequencing methods

	Single	Paired
RCPT/E	73	131
RCPT/B	43	75

Chi-square test: p = 1

c

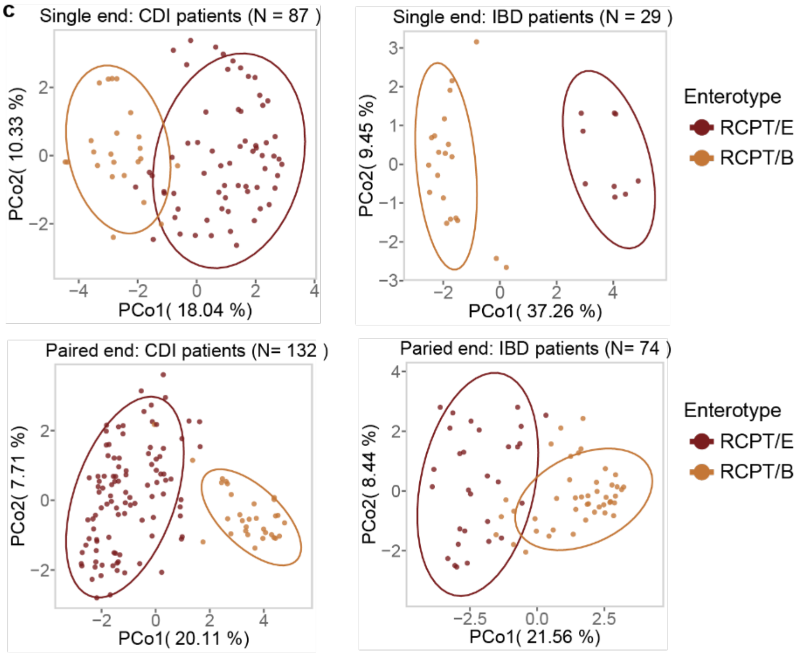

d

DNA extraction

	MoBio	Others
RCPT/E	168	36
RCPT/B	98	20

Chi-square test: p = 1

e

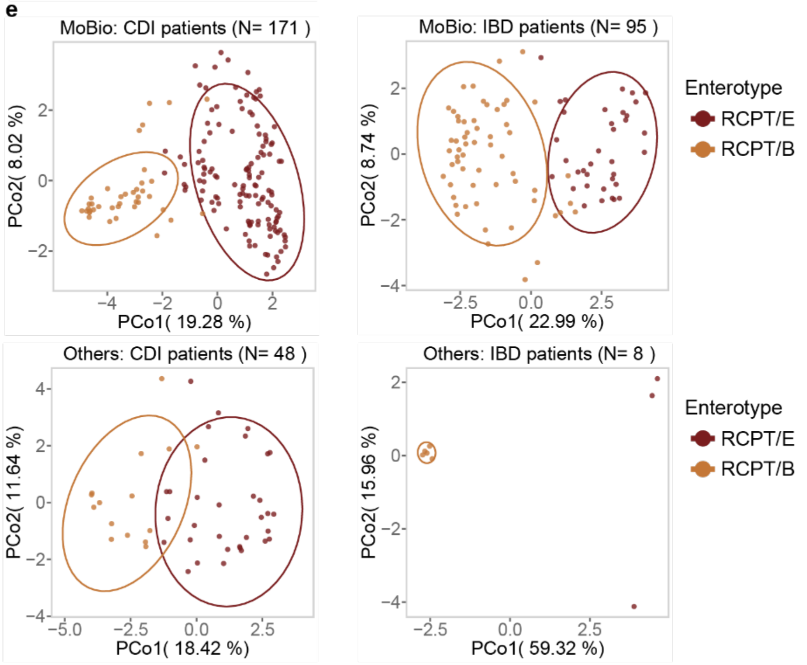

Supplementary Figure S4. Effect of confounders in enterotype clustering. (a) Principal coordinates analysis (PCoA) analysis of samples from different 16S rRNA regions. (b) Sample distribution of two enterotype in different sequencing methods

(single-end / paired-end). (c) Enterotype clustering in samples from different sequencing methods independently. (d) Sample distribution of two enterotype in different DNA extraction methods. (e) Enterotype clustering in samples from different DNA extraction methods independently. “MoBio” represents MoBio DNA extraction kits, “Others” vice versa.

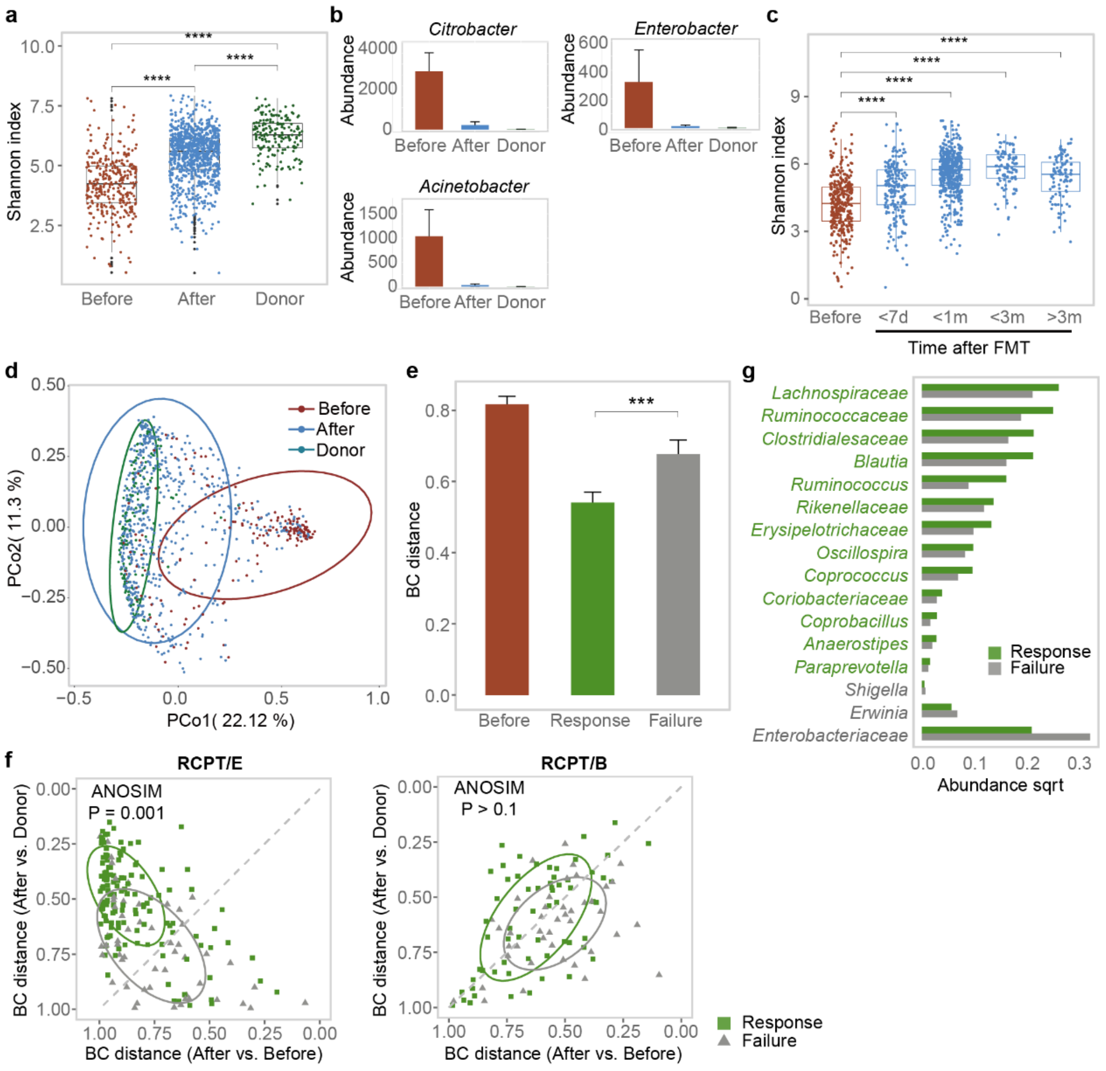

Supplementary Figure S5. FMT outcomes associates with the Bray-Curtis distance of microbiota between donors and recipients. (a) Alpha diversity of recipients and donors during FMT (n = 322). “Before” and “After” represents recipients before and after FMT. Asterisks indicate significance (****p = < 0.0001, ***p = < 0.001). (b) Relative abundance of 3 ET_E-characteristic genera (*Citrobacter*, *Enterobacter*, and *Acinetobacter*) in patients and donors during FMT. Error bar indicates 95% confidence interval. (c) Alpha diversity of recipients over time during FMT. Asterisks indicate significance (****p = < 0.0001, ***p = < 0.001). (d) Bray-Curtis beta-diversity ordination of samples from patients and donors during FMT (number of samples = 1,440). “Before” and “After” represent the recipients before and after FMT. (e) Bray-Curtis distance between donors and recipients with different FMT outcomes (response/failure) (patients before FMT, n = 322; recipients with outcomes, n = 286). (f) Distribution of recipients in different FMT outcome groups (response and failure) in RCPT/E and RCPT/B independently. (g) Mean relative abundance of significantly associated taxa with FMT outcomes (Wilcoxon test, q < 0.05). Family

name represents the genus for f__Family name; g__. The q value represents the p value adjusted by the Benjamini-Hochberg false discovery rate.

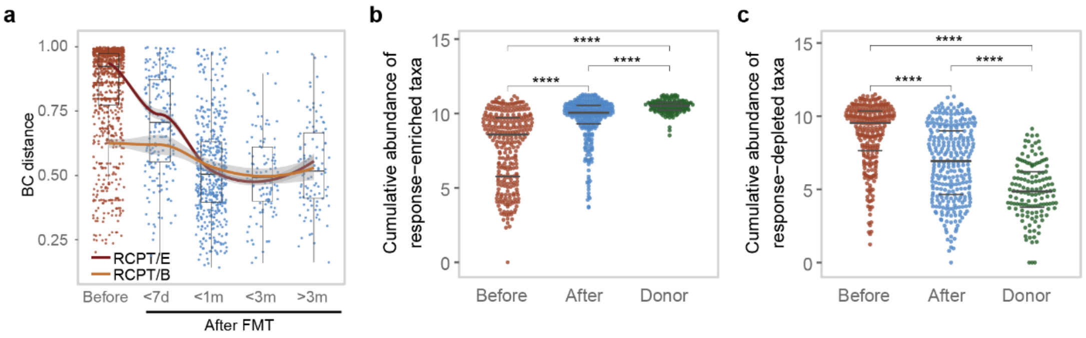

Supplementary Figure S6. FMT outcome was associated with the microbiota distance between recipient and donor in both CDI and IBD. (a) Community variability was determined by the Bray-Curtis (BC) distance over time during FMT. The red and blue dots represent the BC distance between the recipient and its donor before and after FMT. The two lines fit the trends of RCPT/E and RCPT/B, respectively. (b-c) The cumulative abundance of significantly response-enriched or response-depleted taxa in donors and patients before FMT (Wilcoxon test, $q < 0.05$). The q value represents the p value adjusted by the Benjamini-Hochberg false discovery rate. Cumulative abundance was calculated by summing all genera that were significantly enriched (left) or depleted (right) in the response group. Asterisks indicate significance (****$p < 0.0001$, ***$p < 0.001$).

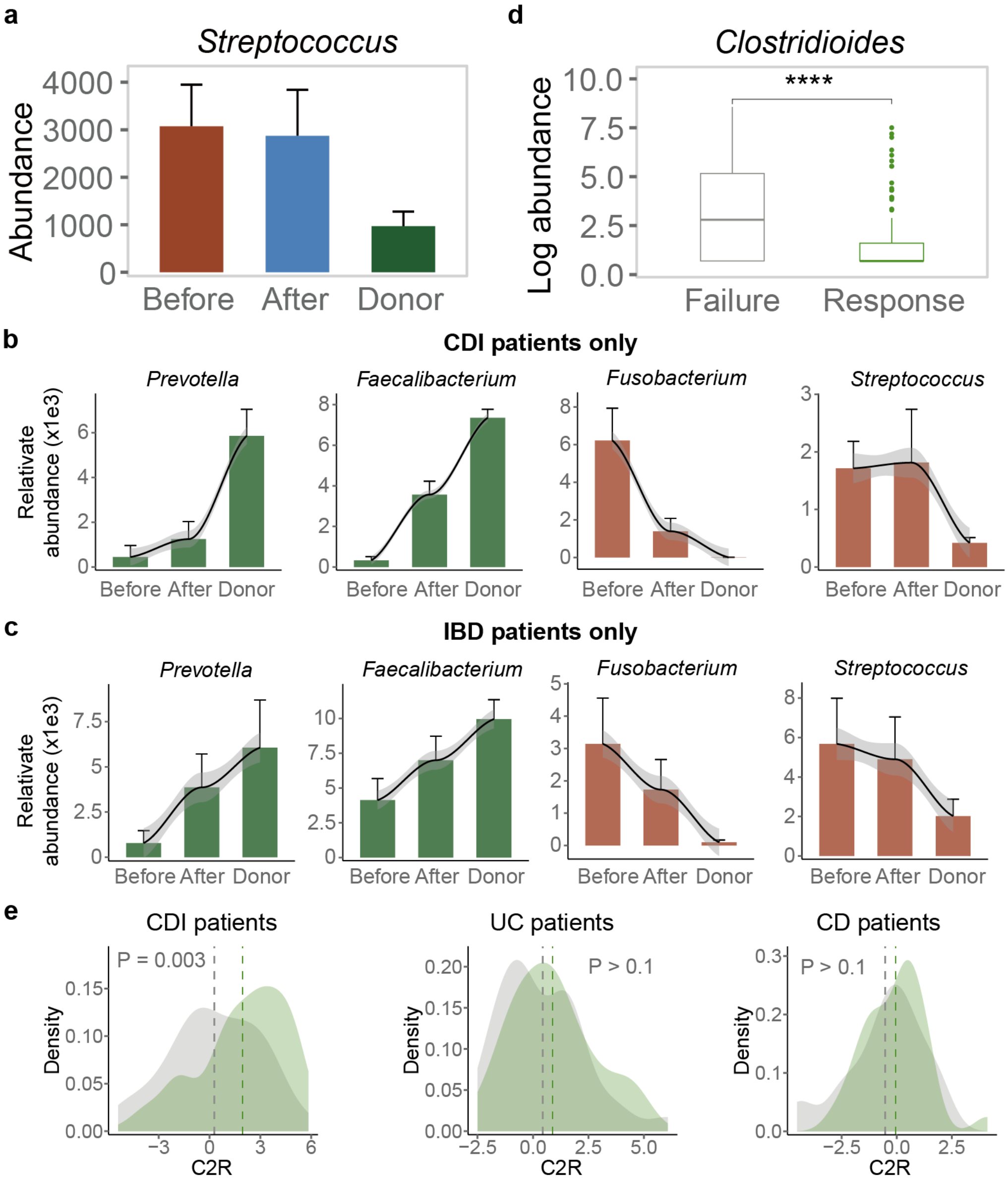

Supplementary Figure S7. Bacterial engraftment in either CDI or IBD recipients. (a) Relative abundance of a resident (*Streptococcus*) during FMT. The error bar indicates the 95% confidence interval. (b) Mean relative abundance of residents and colonizers in CDI recipients and donors during FMT. Colonizer genera, *Prevotella* and *Faecalibacterium*, were shown in the left panel. Resident genera, *Fusobacterium* and *Streptococcus*, were shown in the right panel. (c) Mean relative abundance of residents and colonizers in IBD recipients and donors during FMT. Colonizer genera, *Prevotella* and *Faecalibacterium*, were shown in the left panel. Resident genera, *Fusobacterium* and *Streptococcus*, were shown in the right panel. (d) The relative abundance of a resident (*Clostridioides*) in different outcome groups after FMT. The boxplot center represents median, and the box shows the interquartile range (IQR). Whiskers extend to the most extreme data point <1.5 x IQR. Asterisks indicate significance (****p = <

0.0001, ***p = < 0.001). (e) Distribution of recipients in different FMT outcome groups (response and failure) in either CDI or IBD recipients. From left to right, C2R applied in CDI recipients (left), IBD subtype UC recipients (middle) and IBD subtype CD recipients (right). The two coordinates represent the BC distance between the recipient after FMT and the same recipient before FMT or their donor before FMT, respectively. The green and gray points represent the response and failure of FMT, respectively.

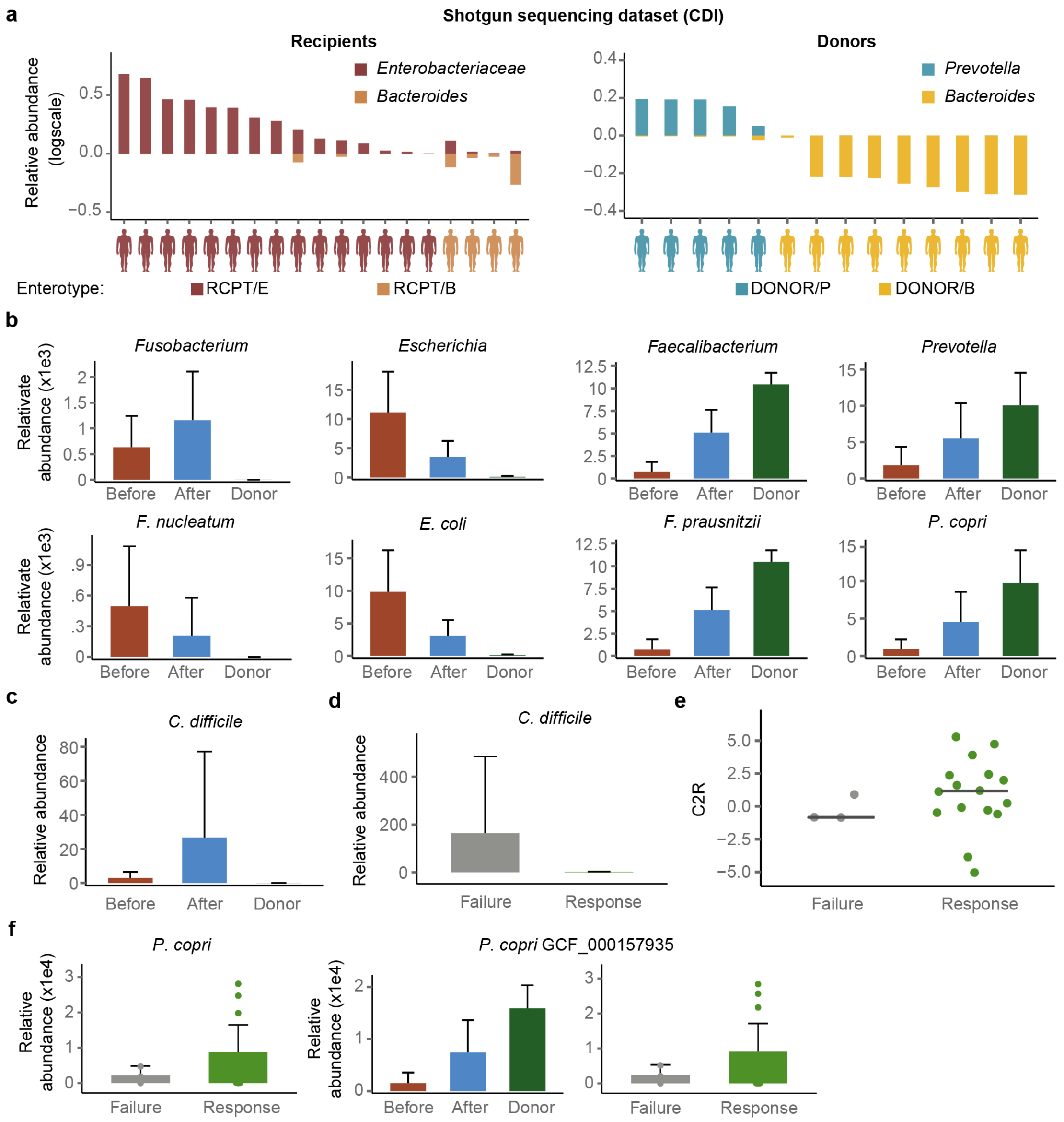

Supplementary Figure S8. Taxa transmission in the shotgun sequencing datasets of 19 CDI recipients. (a) Enterotype classification in the recipients and donors. RCPT/E and RCPT/B were found in the CDI recipients indicated by Enterobacteriaceae and *Bacteroides*; DONOR/P and DONOR/B were found in the corresponding donors indicated by *Prevotella* and *Bacteroides*. (b) Mean relative abundance of residents and colonizers in recipients and donors during FMT. Resident genera (*Fusobacterium* and *Escherichia*) and resident species (*F. nucleatum* and *E. coli*) were shown in the left panel. Colonizer genera (*Faecalibacterium* and *Prevotella*) and colonizer species (*F. prausnitzii* and *P. copri*) were shown in the right panel. (c) Mean relative abundance of residents *C. difficile* in recipients and donors during FMT. (d) Relative abundance of *C. difficile* in the failure and response groups. (e) C2R in the response and failure groups. (f) Left: relative abundance of *P. copri* in the failure and response groups treated with

DONOR/P after FMT. Middle: mean relative abundance of residents *P. copri* GCF_000157935 in recipients treated with DONOR/P and donors during FMT. Right: relative abundance of *P. copri* GCF_000157935 in the failure and response groups treated with DONOR/P after FMT.

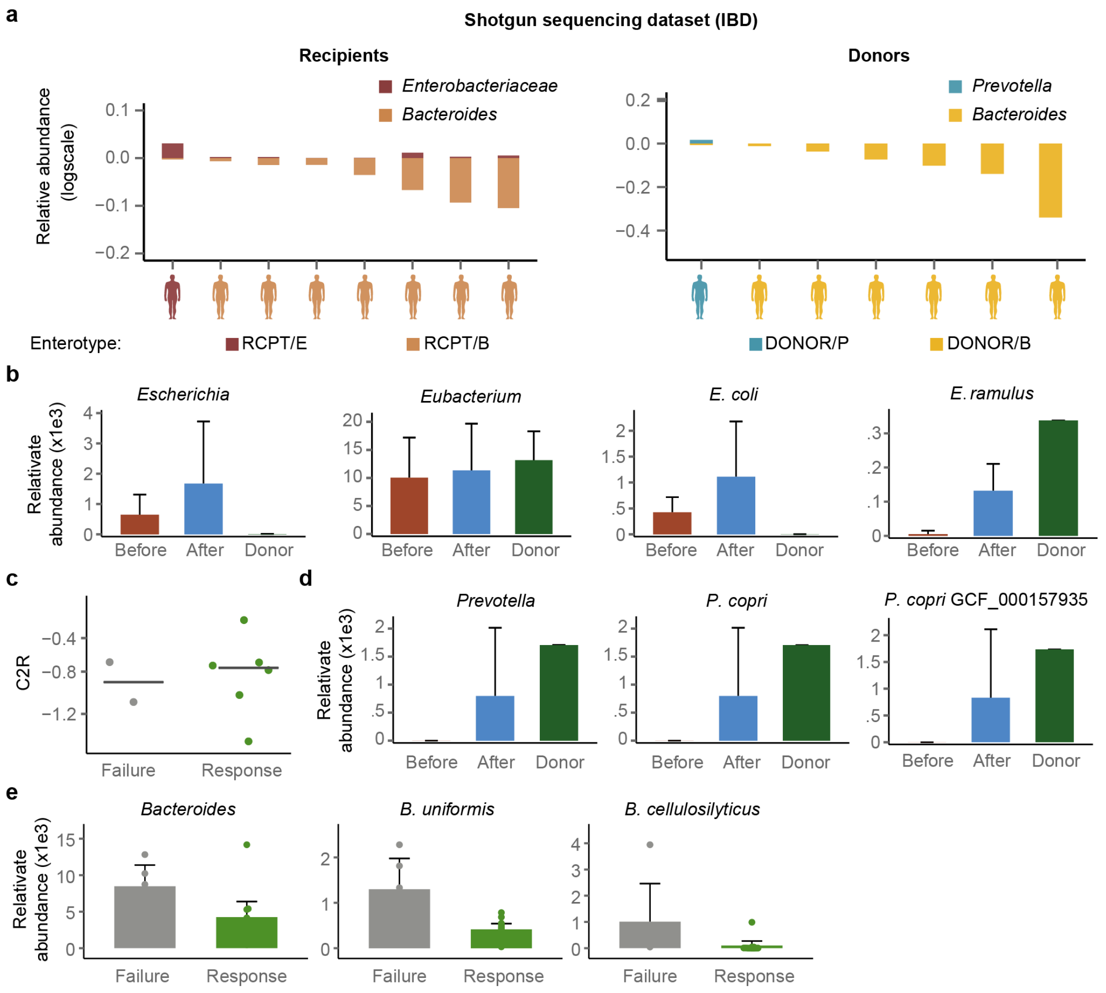

Supplementary Figure S9. Taxa transmission in the shotgun sequencing datasets of 8 IBD recipients. (a) Enterotype classification in the recipients and donors. RCPT/E and RCPT/B were found in the CDI recipients indicated by Enterobacteriaceae and *Bacteroides*; DONOR/P and DONOR/B were found in the corresponding donors indicated by *Prevotella* and *Bacteroides*. (b) Mean relative abundance of residents and colonizers in recipients and donors during FMT. Resident genus (*Escherichia*) and colonizer genus (*Eubacterium*) were shown in the left panel. Resident species (*E. coli*) and colonizer species (*E. ramulus*) were shown in the right panel. (c) C2R in the response and failure groups. (d) Mean relative abundance of colonizer *Prevotella*, *P. copri* and *P. copri* GCF_000157935 in recipients treated with the DONOR/P and donors during FMT. (e) Relative abundance of *Bacteroides*, *B. uniformis* and *B. cellulosilyticus* in the failure and response groups treated with the DONOR/B after FMT.

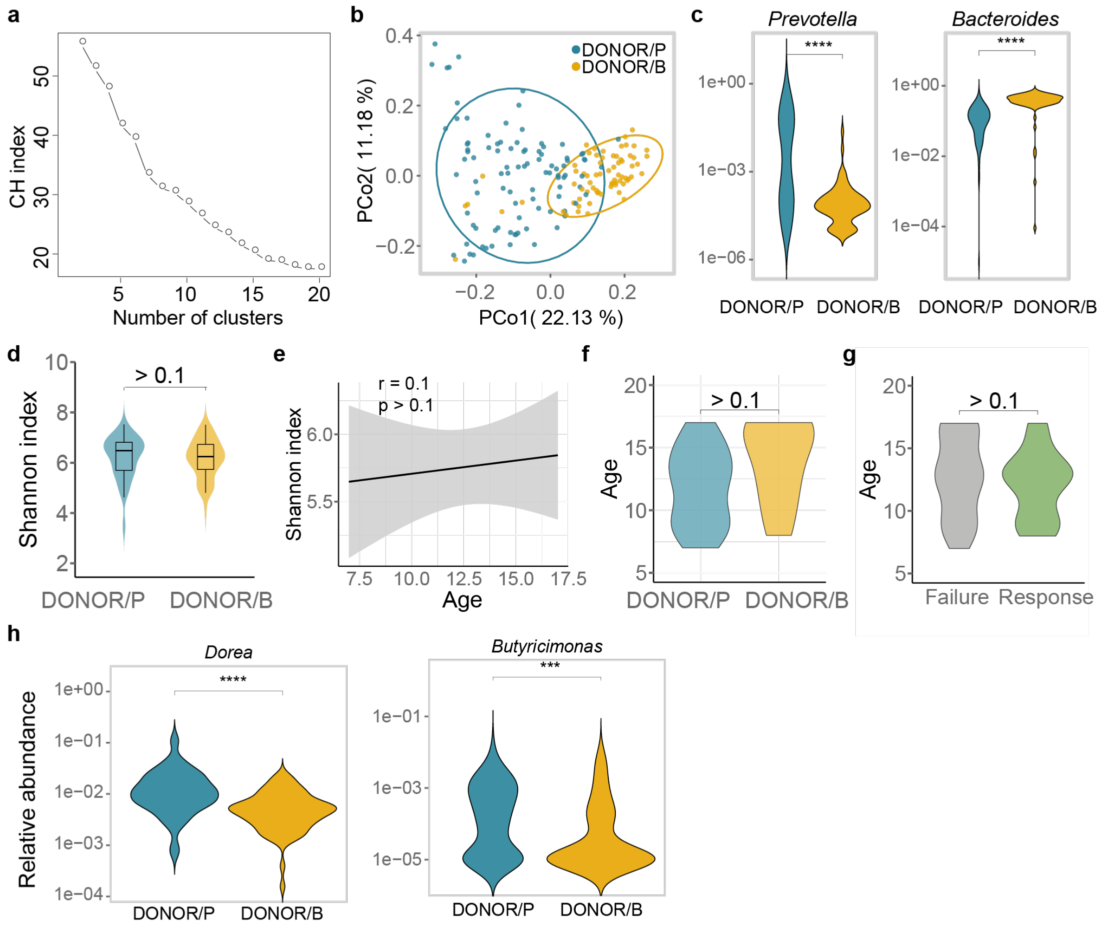

Supplementary Figure S10. Enterotype-based donor-recipient matching contributes to FMT success. (a-c) Left to right, the optimal number of clusters by CH index (a), principal coordinates analysis based on Bray-Curtis dissimilarities (b), and relative abundance of two dominant taxa in donors (c). (d) Alpha diversity in DONOR/P and DONOR/B (Wilcoxon test, $p > 0.10$). (e-g) Effect of donor age in FMT. (e) Correlation between donor age and their gut microbial alpha diversity. (f) Donor age difference between DONOR/P and DONOR/B (Wilcoxon test, $p > 0.10$). (g) Donor age difference between FMT response and failure groups (Wilcoxon test, $p > 0.10$). (h) *Dorea* and *Butyricimonas* were enriched in the DONOR/P compared to the DONOR/B (Wilcoxon test, $p < 0.01$). (e) Schema of how to assign enterotype to a newcomer. The label of enterotype was assigned based on the distance to the medoids of existing clusters.

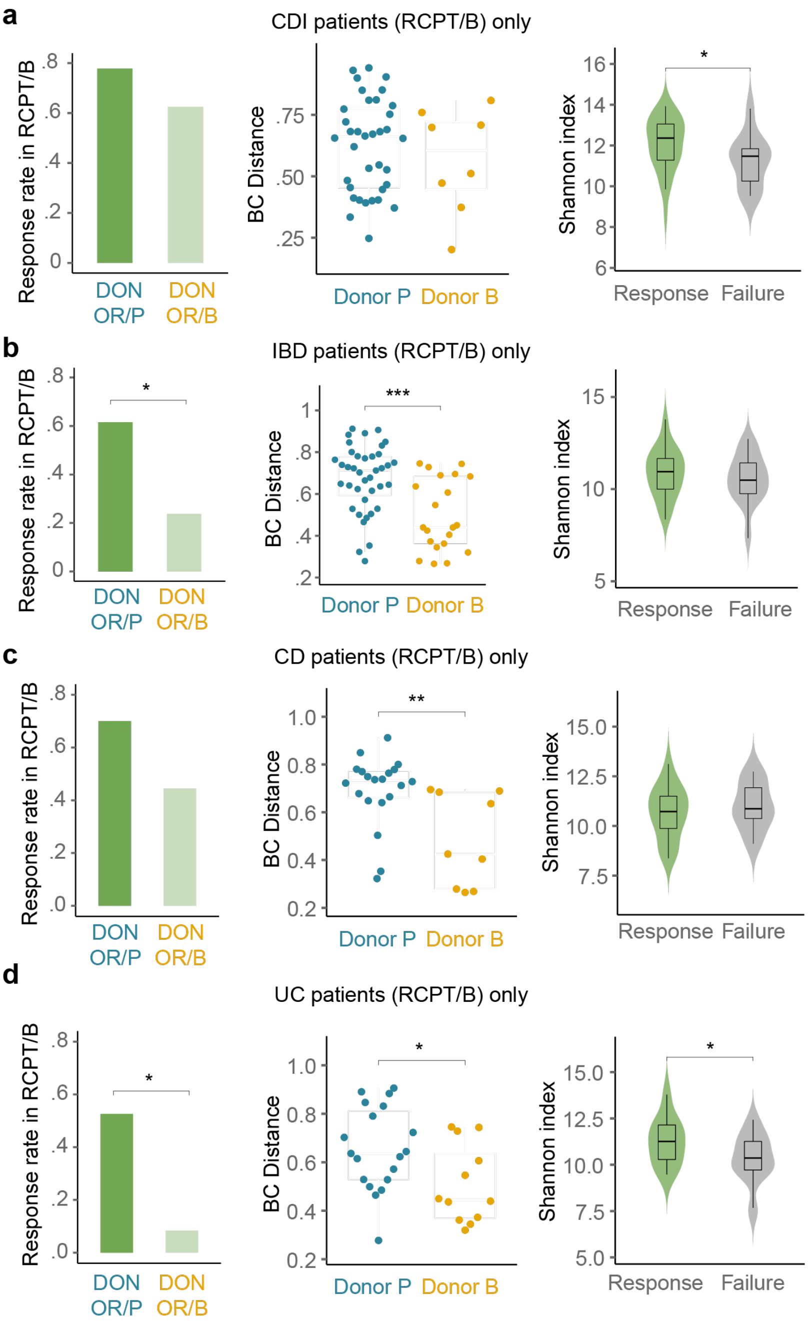

Supplementary Figure S11. Enterotype-based donor-recipient matching contributes to FMT success in either CDI or IBD. Analyses in Fig. 4 were reproduced in CDI patients (a), IBD patients (b), IBD subtype CD patients (b) and IBD subtype UC patients (d). Left: the response rate in corresponding RCPT/B recipients between DONOR/P and DONOR/B (chi-square test, *p < 0.05). Middle: BC distance between RCPT/B patients and donors of DONOR/P or DONOR/B in corresponding disease solely (Wilcoxon test, *p < 0.05). Right: The summed alpha diversity of paired recipient and donor between the response group and the failure group of RCPT/B in CDI corresponding disease solely (Wilcoxon test, ***p < 0.001, **p < 0.01, *p < 0.05).

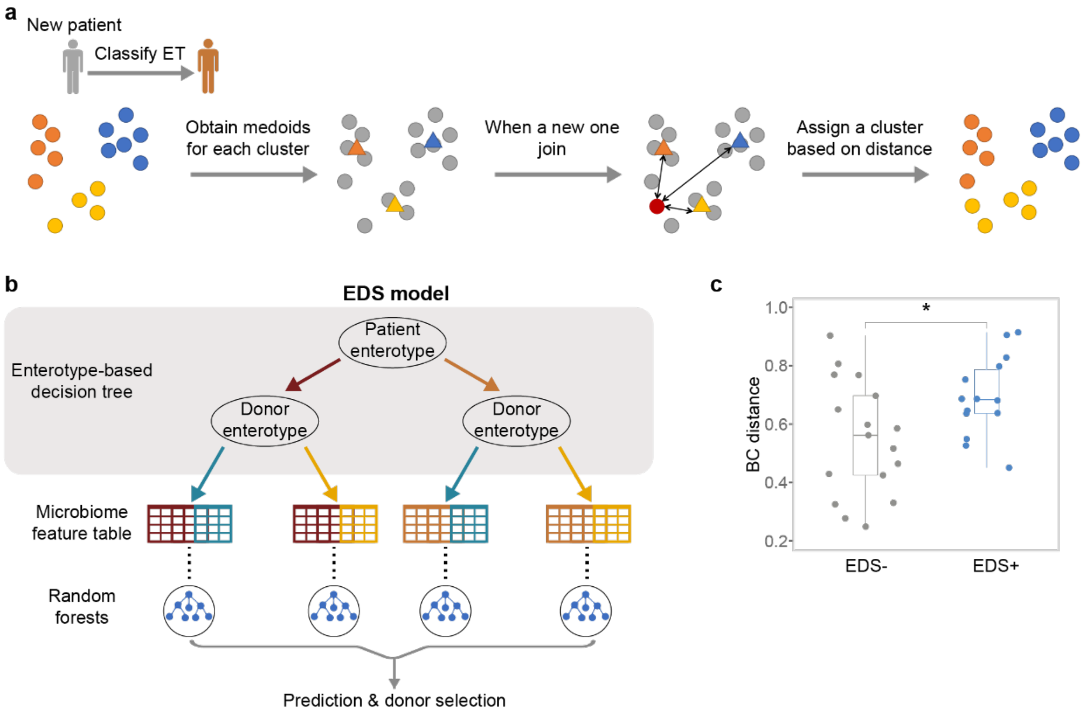

Supplementary Figures S12. Construction and validation of the enterotype-based donor selection model. (a) Enterotype assignment in a new patient. For a new patient that needs FMT, enterotype was assigned based on microbiota distance to two enterotypes' medians. (b) Schema of enterotype-based donor selection (EDS) model for each recipient. Patients and donor pairs were firstly determined by their enterotypes based on enterotype-based decision tree, and then predicted matching degree by random forest classifiers. Random forest classifiers were trained for each enterotype based on features of microbial features, respectively. (c) Bray-Curtis distance between recipients and donors in the EDS+ group and the EDS- group in the validation cohort. Asterisks indicate significance (**p = < 0.01, *p = < 0.05).

Supplementary Tables

Supplementary Table S1: Sample metadata of our recruited discovery cohort

#Patient	Sex	Age	Disease subtype	CD location (Montreal)	UC location (Montreal)	HBI/Mayo score
61	21 females<br>40 males	31.3<br>± 3.1	33 CD<br>28 UC	L1: 5<br>L2: 8<br>L3: 18<br>L1+L4: 2	E3: 23<br>E2: 4<br>E1: 1	10.2<br>± 1.0

Illness degree*	Smoking history**	5-ASA history**	Immunomodulators history**	Corticosteroids history**	Adverse event***
1: 7<br>2: 33<br>3: 21	0: 50<br>1: 11	0: 4<br>1: 56	0: 39<br>1: 22	0: 15<br>1: 46	0: 42<br>1: 19

UC ulcerative colitis, CD Crohn’s disease;

*:1, 2, 3 represent illness degree from mild to severe;

**: 0 represents no corresponding history.

***: 0 represents no adverse event. 1 represents adverse event, including increased stool frequency (n = 9), fever (n = 8) and abdominal pain (n = 5).

Supplementary Table S2: Characteristics of all published discovery cohorts

Accession number	Disease	Abx	Route	Sample size	# FMT	16S region	Reads	References
PRJEB19232	CDI	T	Lower	138	38	V4	Single	[1]
PRJEB19996	CDI	T	Lower	150	4	V4	Single	[2]
PRJNA238042	CDI	T	Mid	40	14	V3-V5	Single	[3]
PRJNA238486	CDI	T	Lower	26	3	V6	Single	[4]
PRJNA296920 and PRJNA311224	CDI	T	Mid	599	105	V5V6	Paired	[5-7]
PRJNA303184	CDI	T	Lower	130	22	V5V6	Paired	[8]
PRJNA353587 and PRJNA3535980	CDI	T	Mid	83	9	V4	Single	[9]
PRJNA384621	CDI	T	Lower	44	6	V4	Paired	[10]
PRJNA414451	CDI	T	Both	37	7	V4	Single	[11]
PRJNA221789	CDI	T	Mid	56	14	V1-V3	Single	[12]
PRJNA419097	CDI	T	Mid	104	16	V4	Paired	[13]
PRJNA380944	IBD	T	Lower	109	21	V4	Single	[14]
PRJNA428898	IBD	F	Mid	35	9	V4V5	Paired	[15]
PRJNA412501	IBD	T	Lower	71	19	V3V4	Single	[16]
PRJDB4959	IBD	F	Mid	40	10	V1V2	Single	[17]

CDI *Clostridioides difficile* infection, IBD inflammatory bowel disease;

Abx: whether use antibiotics before transplantation;

Route: "Lower" means transplantation from down to the lower gut, like colonoscopy; "Mid" means transplantation from up to the mid gut, like endoscopic and nasojejunal tube.

Supplementary Table S3: Negative correlations with [*Clostridium*] (Spearman test, r < -0.1) in donors

Genus	Coefficient
k__Bacteria;p__Bacteroidetes;c__Bacteroidia;o__Bacteroidales;f__Bacteroidaceae;g__Bacteroides	-0.14285
k__Bacteria;p__Bacteroidetes;c__Bacteroidia;o__Bacteroidales;f__Porphyromonadaceae;g__Parabacteroides	-0.14597
k__Bacteria;p__Bacteroidetes;c__Bacteroidia;o__Bacteroidales;f__Prevotellaceae;g__Prevotella	-0.10746
k__Bacteria;p__Bacteroidetes;c__Bacteroidia;o__Bacteroidales;f__Rikenellaceae;g__	-0.20829
k__Bacteria;p__Bacteroidetes;c__Bacteroidia;o__Bacteroidales;f__S24-7;g__	-0.11196
k__Bacteria;p__Bacteroidetes;c__Bacteroidia;o__Bacteroidales;f__[Barnesiellaceae];g__	-0.18038
k__Bacteria;p__Bacteroidetes;c__Bacteroidia;o__Bacteroidales;f__[Odoribacteraceae];g__Odoribacter	-0.13739
k__Bacteria;p__Firmicutes;c__Bacilli;o__Bacillales;f__Staphylococcaceae;g__Jeotgalicoccus	-0.10278
k__Bacteria;p__Firmicutes;c__Clostridia;o__Clostridiales;f__Christensenellaceae;g__	-0.14288
k__Bacteria;p__Firmicutes;c__Clostridia;o__Clostridiales;f__Clostridiaceae;g__Clostridium	-0.10756
k__Bacteria;p__Firmicutes;c__Clostridia;o__Clostridiales;f__Lachnospiraceae;g__Lachnospira	-0.17336
k__Bacteria;p__Firmicutes;c__Clostridia;o__Clostridiales;f__Veillonellaceae;g__	-0.16031
k__Bacteria;p__Proteobacteria;c__Betaproteobacteria;o__Burkholderiales;f__Alcaligenaceae;g__Sutterella	-0.142463377
k__Bacteria;p__Proteobacteria;c__Betaproteobacteria;o__Burkholderiales;f__Oxalobacteraceae;g__Oxalobacter	-0.15321136

Supplementary Table S4: Most important microbial feature used in EDS model, except enterotype.

RCPT/E + DONOR/P	RCPT/E + DONOR/B	RCPT/B + DONOR/P	RCPT/B + DONOR/B
*Devosia*_Recipient	Alpha diversity_Don+Recip	*Epulopiscium*_Recipient	*Haemophilus*_Recipient
*Collinsella*_Recipient	*Acidaminococcus*_Donor	*Adlercreutzia*_Donor	*Streptococcus*_Recipient
*Clostridioides*_Recipient	*Collinsella*_Donor	*Morganella*_Recipient	*Pasteurellaceae*_Recipient
*Megasphaera*_Recipient	*Neisseriaceae*_Recipient	*Weissella*_Recipient	*Clostridium*_Recipient
*Phyllobacteriaceae*_Recipient	*Mogibacteriaceae*_Recipient	*Trabulsiella*_Recipient	Alpha diversity_Donor
*Rhodospirillaceae*_Recipient	*Vagococcus*_Recipient	*Methanobrevibacter*_Donor	*Klebsiella*_Recipient
*Serratia*_Recipient	*Comamonadaceae*_Recipient	*Roseburia*_Recipient	*Lactococcus*_Recipient
*Megamonas*_Donor	*Bulleidia*_Donor	*Actinomyces*_Recipient	*Actinomyces*_Recipient
*Leuconostoc*_Recipient	*Haemophilus*_Donor	*Pasteurellaceae*_Recipient	*Eikenella*_Recipient
*Gemellaceae*_Recipient	*SMB53*_Recipient	*Providencia*_Recipient	*Akkermansia*_Donor

Supplementary Table S5: Sample metadata of our recruited validation cohort

#Patient	Sex	Age	Disease subtype	Illness degree*	Course of disease
42	16 females<br>26 males	35.5<br>± 4.7	9 CD<br>33 UC	1: 5<br>2: 23<br>3: 14	4.8<br>± 1.6

UC ulcerative colitis, CD Crohn's disease;

*:1, 2, 3 represent illness degree from mild to severe;

References

1. Khanna, S., et al., *Changes in microbial ecology after fecal microbiota transplantation for recurrent C. difficile infection affected by underlying inflammatory bowel disease.* Microbiome, 2017. **5**(1): p. 55.
2. Weingarden, A., et al., *Dynamic changes in short- and long-term bacterial composition following fecal microbiota transplantation for recurrent Clostridium difficile infection.* Microbiome, 2015. **3**: p. 10.
3. Seekatz, A.M., et al., *Recovery of the gut microbiome following fecal microbiota transplantation.* mBio, 2014. **5**(3): p. e00893-14.
4. Shankar, V., et al., *Species and genus level resolution analysis of gut microbiota in Clostridium difficile patients following fecal microbiota transplantation.* Microbiome, 2014. **2**(13): p. 13.
5. Staley, C., et al., *Community dynamics drive punctuated engraftment of the fecal microbiome following transplantation using freeze-dried, encapsulated fecal microbiota.* Gut Microbes, 2017. **8**(3): p. 276-288.
6. Staley, C., et al., *Gut-sparing treatment of urinary tract infection in patients at high risk of Clostridium difficile infection.* J Antimicrob Chemother, 2017. **72**(2): p. 522-528.
7. Staley, C., et al., *Predicting recurrence of Clostridium difficile infection following encapsulated fecal microbiota transplantation.* Microbiome, 2018. **6**(1): p. 166.
8. Kelly, C.R., et al., *Effect of Fecal Microbiota Transplantation on Recurrence in Multiply Recurrent Clostridium difficile Infection: A Randomized Trial.* Ann Intern Med, 2016. **165**(9): p. 609-616.
9. Zuo, T., et al., *Bacteriophage transfer during faecal microbiota transplantation in Clostridium difficile infection is associated with treatment outcome.* Gut, 2018. **67**(4): p. 634-643.
10. Seekatz, A.M., et al., *Restoration of short chain fatty acid and bile acid metabolism following fecal microbiota transplantation in patients with recurrent Clostridium difficile infection.* Anaerobe, 2018. **53**: p. 64-73.
11. Camacho-Ortiz, A., et al., *Randomized clinical trial to evaluate the effect of fecal microbiota transplant for initial Clostridium difficile infection in intestinal microbiome.* PLoS One, 2017. **12**(12): p. e0189768.
12. Song, Y., et al., *Microbiota dynamics in patients treated with fecal microbiota transplantation for recurrent Clostridium difficile infection.* PLoS One, 2013. **8**(11): p. e81330.
13. Zuo, T., et al., *Gut fungal dysbiosis correlates with reduced efficacy of fecal microbiota transplantation in Clostridium difficile infection.* Nat Commun, 2018. **9**(1): p. 3663.
14. Goyal, A., et al., *Safety, Clinical Response, and Microbiome Findings Following Fecal Microbiota Transplant in Children With Inflammatory Bowel Disease.* Inflamm Bowel Dis, 2018. **24**(2): p. 410-421.
15. Li, P., et al., *Timing for the second fecal microbiota transplantation to maintain the long-term benefit from the first treatment for Crohn's disease.* Appl Microbiol Biotechnol, 2019. **103**(1): p. 349-360.
16. Mintz, M., et al., *Longitudinal microbiome analysis of single donor fecal microbiota*

transplantation in patients with recurrent Clostridium difficile infection and/or ulcerative colitis. PLoS One, 2018. **13**(1): p. e0190997.

17. Mizuno, S., et al., *Single fecal microbiota transplantation failed to change intestinal microbiota and had limited effectiveness against ulcerative colitis in Japanese patients.* Intest Res, 2017. **15**(1): p. 68-74.
